# Silane Coatings for Corrosion and Microbiologically Influenced Corrosion Resistance of Mild Steel: A Review

**DOI:** 10.3390/ma15217809

**Published:** 2022-11-05

**Authors:** Saad Al-Saadi, R. K. Singh Raman

**Affiliations:** 1 Department of Mechanical and Aerospace Engineering, Monash University, Clayton, VIC 3800, Australia; 2 Department of Chemical and Biochemical Engineering, Monash University, Clayton, VIC 3800, Australia

**Keywords:** silane coatings, mild steel, microbiologically influenced corrosion, silane hydrolysis and condensation

## Abstract

Mild steel continues to be the most extensively used construction material in several industries and constructions. However, corrosion of mild steel in aggressive environments is a major concern. Under the tremendously increasing demand for improving the coatings strategies because of the environmental concerns due to some of the traditional coatings, silane pre-treatments have been emerging as one of the effective solutions, among other strategies. Different approaches, such as adding particles of metal oxide (such as SiO_2_, ZrO_2_, Al_2_O_3_, TiO_2_ and CeO_2_), incorporating plant extracts and impregnating 2D materials into the coatings, have been employed for durable corrosion resistance, including for mitigating enhanced corrosion due to the presence of bacteria. This review discusses the critical mechanistic features of silane coatings such as the role of hydrolysis and condensation in the bonding of silanes with metal surfaces. The factors that influence the performance of the silane coatings for corrosion resistance of mild steel are discussed. In particular, this review provides insight into silane coatings for mitigating microbiologically influenced corrosion (MIC) of mild steel.

## 1. Introduction

Organofunctional silicones, also known as silanes, are hybrid compounds of silica and organic materials related to resins; therefore, they can be used as coupling agents to improve bonding of inorganic substrates such as metals to organic resins [1,2]. The efficiency of silanes is attributed to their function as coupling agents, as they work as a chemical bridge whereby one part of the molecule attaches to the inorganic substrate (such as glass, metal or mineral) and another part ties to the adhesive, coating or polymer [1,3,4,5]. The main applications of these chemicals have been at glass/paint, metal/paint and metal/rubber interfaces [6].

The organofunctional silanes which are used as adhesion promoters or coupling agents have a general structure of X_3_Si(CH_2_)_n_Y, where the attachment of silanes to surfaces is established through the hydrolysable alkoxy group (X), where X can be methoxy (OCH_3_), ethoxy (OC_2_H_5_) or acetoxy (OCOCH_3_) [7,8]. The enhancement of the strength of the silane–paint polymer interface is the primary function of the functional group Y through the formation of chemical bonds [9]. The most important step to form the organofunctional group of silanes is the addition of silicon hybrids to substituted olefins and acetylenes according to the reaction [10]:

(1) $$X_{3}SiH+CH_{2}=CH-R-Y\;\;\to \;X_{3}SiCH_{2}CH_{2}RY$$

The organosilanes that have an additional Si atom linked to the trialkoxy group (X_3_) on the other side of the functional group (*Y*) and have the general structure *X*_3_*Si*(*CH*_2_)*_n_Y*(*CH*_2_)*_n_SiX*_3_ are called bis-silanes. The functional group *Y* can be, for example, an amine group or a chain of sulfur atoms. The non-functional silanes (i.e., the silanes without the functional group) have been investigated for their use as cross-linking agents, and they do not provide good paint adhesion [11,12]. For many decades, chromate conversion coatings have been used effectively as a corrosion inhibitor as well as to create an interface compatible with a wide range of paints. Cr(VI)-based treatments have historically played a very important role in the aircraft, marine, automotive and civil construction industries for corrosion resistance of different metals and alloys [13]. However, there are increasing regulatory restrictions on the use of chromate compounds because they are harmful and hazardous to health and the environment, including the serious concern of the carcinogenic effect of hexavalent Cr. Tsapakos et al. [14] have reported the chromium (III) produced as result of Cr(VI) reduction may be directly involved in mediating cross-linking of cell macromolecules, such as DNA and proteins, leading to mutagenic and carcinogenic activities of chromium. As a result, non-toxic alternatives to chromate treatments have attracted remarkable research attention. Organosilanes, which are one such alternative, are attractive for several industrial applications since they can be used not only as adhesion promoters for a wide range of paints but also as corrosion barriers for several metals and alloys [12,15]. Functional materials processing of silanes and organosilanes falls in the category of “sol-gel”. The final characteristics of the synthesized protective coatings depend on their precursor chemistry, temperature, pH of the solution, molar ratios of reactants, solvent composition, water and catalyst [16]. Depending on the silane precursors used for syntheses, different sol–gel materials are produced that possess unique functionalities for specifically modifying the surface properties required for specific applications [16]. In addition, silane treatments are an easy application process, relatively inexpensive and an environment-friendly option [17].

Mild steel is the most frequently used construction material in many industries due to its excellent mechanical properties and low cost [18]. However, it is prone to surface degradation when exposed to corrosive environments. Silane coatings are emerging as one of the environment-friendly approaches to circumvent the corrosion of metallic materials including mild steels. This review first presents an overview of the considerable knowledge of silane hydrolysis and the mechanism of their bonding with the metallic substrates as well as of the comprehensive knowledge of the silane coatings for corrosion resistance of mild steel, with a particular emphasis on the role of silane coating for the mitigation of microbiologically influenced corrosion (MIC).

## 2. Hydrolysis and Condensation of Organosilanes

**Figure 1:**
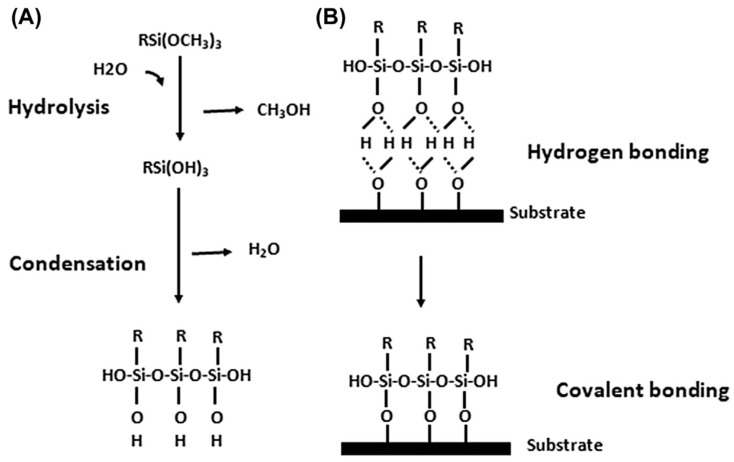
Hydrolysis and condensation of alkoxysilane and bonding of silanol to the metal substrate. (**A**) Hydrolysis and condensation to form oligomers in the silane solution and (**B**) adsorption to an inorganic substrate (such as ceramics or surface oxide layers on metals) by hydrogen bonding and then covalent bonding to the substrate by a condensation reaction with hydroxyl groups [19,20,21].

The silanes used for improvement of adhesion and surface modification are usually alkoxysilanes, which are hydrolyzed before or during application and attachment processes. Such hydrolysis involves a complex cascade of reactions. Figure 1 illustrates a simplified view of the reaction cascade [19,20,21]. Because water and alkoxysilanes are immiscible, solvents such as alcohol are normally used as a homogenizing medium [22,23,24]. The hydrolysis reaction can be expressed as:

(2) $$R^{/}-{\left(CH_{2}\right)}_{n}-Si-{\left(OR\right)}_{3}+H_{2}O\;↔\;R^{/}-{\left(CH_{2}\right)}_{n}-Si-{\left(OH\right)}_{3}+3ROH$$

The subsequent reaction is the condensation of the trihydroxysilane, as expressed in the following equation:

(3) $$R^{/}-{\left(CH_{2}\right)}_{n}-Si-{\left(OH\right)}_{3}\;↔\;R^{/}-{\left(CH_{2}\right)}_{n}-Si{\left(OH\right)}_{2}-O-Si{\left(OH\right)}_{2}-{\left(CH_{2}\right)}_{n}-R^{/}+H_{2}$$

**Figure 2:**
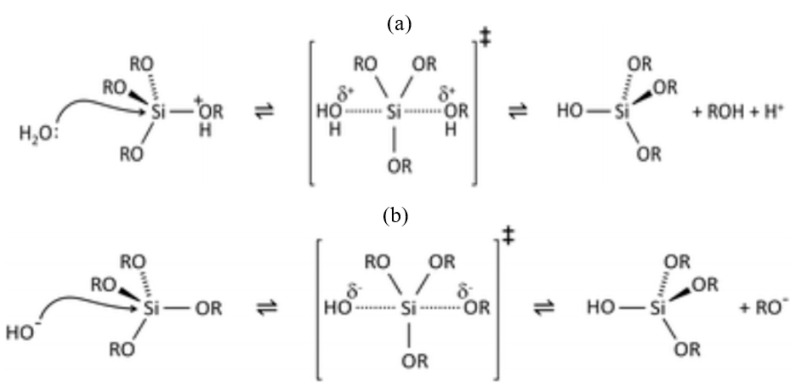
(**a**) Acid- and (**b**) base-catalyzed hydrolysis of silicon alkoxides. The alkoxy group (OR) (such as methoxy (OCH_3_), ethoxy (OC_2_H_5_) or acetoxy (OCOCH_3_)) is replaced with the hydroxyl with a pentacoordinate transition state [26].

**Figure 3:**
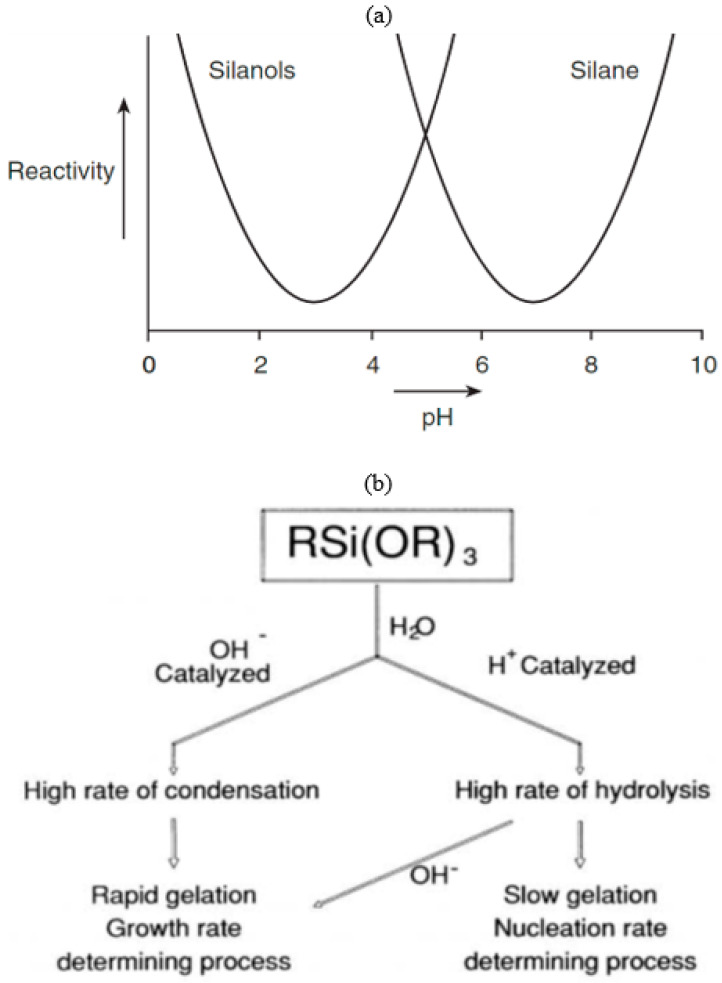
(**a**) Reactivity of silane(s) and silanols. Silanols are most stable at pH around 3, and their reactivity increases when pH is lower than 1.5 or higher than 4.5 [27]. (**b**) Effect of pH on alkoxysilane hydrolysis. The gross changes in silane condensation and polymerization in an acid vs. base condition [19].

The majority of silanes (with the exemption of aminosilanes) are used for surface treatments under acid-catalyzed conditions, where the rate of hydrolysis is considerably greater than that under the base-catalyzed hydrolysis condition, and it is minimally affected by other carbon-bonded substituents [19]. The hydrolysis is headed by protonation of the alkoxy group [19,25]. In both acid- (Figure 2a) and base-catalyzed (Figure 2b) systems, during hydrolysis, the alkoxy group is replaced with the hydroxyl with a pentacoordinate transition state [26]. The influence of pH on the stability of the alkoxysilanes and the formed silanol’s stability are different [27]. Silanols are most stable at pH around 3, and their reactivity increases when pH is lower than 1.5 or higher than 4.5 (Figure 3a) [27]. The dependency of the hydrolysis and condensation of alkoxysilanes on the pH is different, though acids and bases catalyze both processes [28]. When the reactions are base-catalyzed, gelation is fast due to the high rate of condensation, whereas, in acid-catalyzed reactions, the rate of hydrolysis is high with slow gelation [28]. Danks et al. [26] reported that the hydrolysis step (acid/base) depends on the stability of the transition state, which is dictated by the relative electron-withdrawing or donating power of –OH versus –OR groups. As a result, the subsequent hydrolysis steps become progressively slower in acidic conditions and faster under the basic conditions.

Unless the hydrolysis or the condensation is suppressed by specific conditions, both reactions will carry on simultaneously; for instance, suitable solvents can be used to prevent precipitation or gelation by slowing down or even halting the condensation [28]. Figure 3b shows the overall changes in silane condensation and polymerization in an acid versus base condition [19].

Savard et al. [29] studied the hydrolysis and condensation of γ-methacryloxypropyltrimethoxy silane in aqueous solutions and have confirmed the rates of hydrolysis and condensation to depend on the pH strongly. For example, they noted that about 25% of the initial quantity of silane was precipitated at pH = 7, whereas it was difficult to detect any insoluble in an acidic environment at pH = 2.7. For the same silane, Beari et al. [30] found that the initial concentrations of the generated silanetriol were 88% at pH 4 and 95% at pH 6 after 1.5 and 2.5 h, respectively. The concentrations decreased to below 15% at pH 4 in 74 h and to below 35% at pH 6 in 188 h. On the contrary, the concentrations of active silanol were more than 60% in both cases (i.e., on average, about two hydroxyl functions per silicon atom), confirming the reactivity of the formulation. The hydrolysis of methacryloxypropyltrimethoxy silane happened much more rapidly in acidic solution, but the yield of active silanols was higher at neutral pH, and the tendency to oligomerization was suppressed.

Cihlář [31] investigated the effect of pH and catalysts on the hydrolysis and condensation of tetraethoxysilane in a water–ethanol solution using chromatography, potentiometry and gelation tests. Both strong acids (HCl, HClO_4_, HNO_3_, H_2_SO_4_ and p-toluenesulphonic acid) and weak acids (Cl_3_CCOOH, (COOH)_2_, ClCH_2_COOH, CH_3_COOH and HCOOH) and LiOH were used as catalysts. The hydrolysis is catalyzed by acids as well as by bases. In the acidic region, the hydrolysis constant decreased as pH increased, with the minimum at a pH around 7. The condensation reaction that is catalyzed by acid as well as base is slowest at a pH of about 2.

Pu et al. [28] studied the hydrolysis and condensation kinetics of bis-triethoxysilyl ethane (BTSE) in a water–ethanol solution. The FTIR absorbance of Si-O-C group decreased with increasing duration of hydrolysis, as a result of the substitution of ethoxy groups of BTSE by OH groups. The minimum rate of hydrolysis was detected at pH = 7 (i.e., the rates increased both at pHs lower and higher than 7). They also found the hydrolysis to be much faster, in comparison with the condensation, at pH lower than 4.5, whereas the condensation was faster in the pH range of 4.5–9.0. At pH near 4.5, however, the difference between the hydrolysis and condensation rates tended to be small.

Premachandra et al. [32] investigated the dependence of hydrolysis and condensation kinetics of γ-ureidopropyltrimethoxysilane on the pH in water–methanol by FTIR spectroscopy. They found the silane to be most stable around pH 7.73, where the rate of the first hydrolysis step reached the minimum. In this water–alcohol system, the second and third hydrolysis steps were suppressed at most pHs, because the equilibrium between methanol and silane-bonded methoxy groups was quickly attained. The rates of the condensation reactions are higher at higher pH (e.g., the rates were much higher at pHs 8.97 and 9.87 than at pH 4.87). Silanols and water seemed to reach the equilibrium after sufficient time that was governed by the pH, leading to a constant number of siloxane bonds. The study also concluded that a solution of silane in the water–methanol system around pH 4.87 is rich in silanols due to the fast hydrolysis of methoxy groups and the relatively high stability of the formed silanols around this pH.

The hydrolysis of several alkyl-substituted alkoxysilanes and an antimicrobial quaternary ammonium silane was investigated in water–acetone solvents by FTIR spectroscopy [33]. FTIR spectra of trimethylmethoxysilane in 10% (*w/w*) water in acetone showed, at the initial stages, bands for the asymmetric stretch (1083 cm^−1^) and the symmetric stretch (865 cm^−1^) for Si-O-C methoxy and a water band. After considerable duration, which depends on the solution pH, these bands disappeared and were replaced by the C-O stretch of methanol (1031 cm^−1^) and the Si-OH stretch of the silanol (896 cm^−1^). Such behavior is attributed to the completion of hydrolysis of the methoxy group, as the silanol band is reduced and the siloxane asymmetric stretch appears. The rate of acid-catalyzed hydrolysis of trimethylmethoxysilane in 10% water–acetone was found to be much greater than the rate of condensation of siloxane bonds. A significant condensation is noticed but only after comprehensive hydrolysis. For other alkyltrimethoxysilanes (methyl-, ethyl-, propyl- or butyl-trimethoxysilane), as the alkyl group becomes larger, the acid catalysis becomes less effective. For silane containing a quaternary ammonium chloride group, the FTIR spectroscopy showed very little hydrolysis even after several hours, as the methoxy group was still detected at pH ≈ 5.5. When the pH was adjusted to 2.7, the intensity of the Si-O-C band decreased as a result of extensive hydrolysis, as evidenced by the emergence of the methanol band at 1030 cm^−1^ and the silanol band at 920 cm^−1^. Rapid and extensive condensation occurred when pH was adjusted to 7.0.

Issa et al. [34] investigated the hydrolysis mechanism of 3-cyanopropyltriethoxysilane (CTES). They found that the activation energy (∆G^ǂ^) of CTES hydrolysis in methanol and ethyl acetate resulted from an intermediate step, i.e., re-esterification in the case of methanol and hydrolysis of ethyl acetate at high temperatures, to produce acetic acid and ethanol and autocatalysis by acetic acid.

Paquet et al. [35] investigated the kinetics of the hydrolysis and self-condensation reactions of 3-(2-amino-ethylamino)propyl-trimethoxysilane in pure water and ethanol–water solution, using in situ 29Si NMR spectroscopy. During the early stages of the reaction, the reactivity of the silane was strongly affected by the pH of the hydrolysis environment. The silanols produced upon hydrolysis of silane water were much more stable than those produced by hydrolysis in ethanol–water; the latter was vulnerable to self-condensation, forming a siloxane bridge. They conclude that the relative ratio of silanols in water is influenced by pH that is consistent with the established role of acidic media in suppressing self-condensation.

In summary, the hydrolysis and condensation of silanes are crucial for their adhesion to metal surfaces. The pH of the environment is the most influential factor in silane hydrolysis and condensation processes. In the acidic environment (2 < pH < 4.5), stable silanols are produced. However, the condensation is facilitated in the basic conditions.

## 3. Mechanism of Silane Bonding with Metal

Usually, silanes are stored in a non-hydrolyzed state, and in most cases, they are hydrolyzed just before their use [36]. For bonding of silanes to a metallic substrate, the main purpose of hydrolysis is to generate active and sufficient silanols. The bonding is profoundly dictated by the pre-treatment of the metal surface. When silane is hydrolyzed in water or water + alcohol mixture, alkoxy groups convert to hydrophilic silanol groups (-SiOH) [6,37,38]. This conversion is profoundly governed by the nature of the organofunctional group on silicon and the pH of the solution [39].

**Figure 4:**
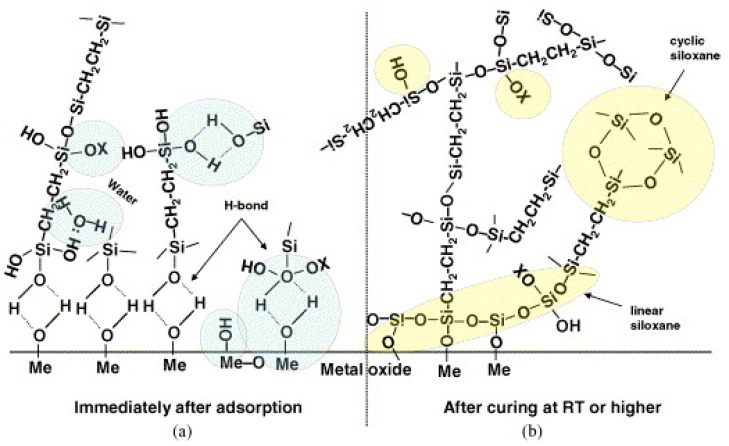
Simplified schematic of bonding mechanism between silane molecules and metal surface hydroxide layer: (**a**) before condensation: hydrogen-bonded interface; (**b**) after condensation: covalent-bonded interface [42].

The silane molecules need to be hydrolyzed to produce a sufficient number of silanol groups in each silane molecule that facilitate bonding with metal surfaces upon subsequent condensation between silanol groups (-SiOH) and metal surface hydroxyls (MeOH), as well as among silanol groups themselves [40], as shown in Figure 1. Metallo-siloxane bonds (Me-O-Si) formed as a result of the subsequent condensation (i.e., curing or drying process) of -SiOH and MeOH groups by releasing water, and a siloxane network (Si-O-Si) formed due to the reaction of silanols among themselves [11,41]. The as-formed Si-O-Si network becomes very hydrophobic if one of the substituents on the Si atom is a carbon atom [37]. For its hydrophobic nature, the formation of siloxane network -Si-O-Si- is crucial in corrosion protection. Figure 4 presents the simplified schematic of the mechanism of bonding between silane molecules and metal’s surface hydroxide layer [42].

Getting et al. [43] investigated the interfaces of abraded mild steel surfaces and various types of silane-based primers, using X-ray photoelectron spectroscopy (XPS) and static secondary ion mass spectroscopy (SSIMS) techniques to ascertain the bonding mechanism between primer and metal. The presence of SiO_2_H^−^, SiOH^+^ and SiO_2_ radicals was attributed to the occurrence of polymerization that produces a polysiloxane structure on the metal substrate. The presence of FeSiO^+^ originated from the surface iron oxide, which also provides strong evidence for the formation of a chemical bond, possibly the metallo-siloxane (Fe-O-Si) bond, as seen in Figure 1. However, no such radicals were detected when styrene-functional amine hydrochloride silane and γ-aminopropyltriethoxy silane were used. Van Ooij and Sabata [44] characterized the films of N-[2-(vinylnenzylamino)-ethyl]-3-aminopropyltrimethoxysilane and γ-aminopropyltriethoxy silane on zinc and steel substrates, respectively, by time-of-flight SIMS (TOFSIMS) and XPS techniques. For the film deposited on zinc surface and not subjected to curing, the high resolution TOFSIMS showed that typical Si peaks were subdued; instead, several high-intensity N peaks appeared. Accordingly, it was proposed that silanols are mainly absorbed by the zinc surface and the aromatic vinyl-benzyl groups are oriented away from the surface. In addition, no siloxane formation could be detected in the spectra. In fact, even after curing, there was only a slight increase in siloxane peaks. The TOFSIMS of γ-APS film deposited at pH 10.5 on an alkali cleaned mild steel showed several peaks for Si and N (without any peak for C) or peaks with Si and two N atoms. The presence of such peaks is due to the silane dimers or oligomers in which a –SiOH group of one molecule is linked with an –NH_2_ group of another molecule. Such internal acid–base interaction can happen in the solutions at a high pH since the –SiOH group becomes somewhat ionic. In fact, such processes involve –SiOH and NH_2_ groups at the metal surfaces if they are adsorbed at adjacent surface sites. When the silane was deposited from a solution of lower pH (e.g., 8.0), the peaks related to siloxane increased, and an obvious increase in the protonation of amino groups occurred. Silane molecules have been suggested to form covalent bond with iron.

Quinton et al. [45] found that the behavior of deposition of propyltrimethoxysilane (PTMS) on iron oxide is similar to that on aluminum oxide. For the silane-coated aluminum, the variation of the mass 28 peak (Si^+^) in SIMS was considerably different from that of the mass 71 peak (Si-O-Al). The intensity of mass 71 peak (Si-O-Al) for PTMS film on aluminum surface reached the maximum when a 0.75% silane concentration was used. Then, the intensity decreased to an asymptotic minimum level at 12% silane concentration. Because of the oscillatory nature of adsorption of PTMS on a number of metal oxide surfaces, including iron oxide, the data showed a fluctuation in the intensity of the mass 28 (Si^+^) peaks with increasing silane concentration. They also found that the maximum intensity of the mass 100 peak (SiOFe^+^) was at the same concentration as the first maximum of the mass 28 (Si^+^) peak. However, the rise of the mass 28 peak to an asymptotic level was at the maximum silane concentration (12%), whereas the intensity of the mass 100 peak decreased as the silane concentration increased. In another study, TOFSIMS of a non-organofunctional silane (i.e., BTSE) deposited on Al and Zn metal and Al-43.4Zn-1.6Si alloy also indicated the existence of metal–oxygen–silicon type of ion fragments which gave evidence for a chemical interaction between silane and the metal substrates [46].

Petrunin et al. [47] studied the mechanism of the formation of alkoxysilane adsorption layers from the vapor phase on a pure aluminum surface and the effect of this layer on corrosion resistance. They found that the first monolayer is adsorbed irreversibly with van der Waals forces. Subsequently, in the presence of water, covalent bonds develop between the silane and metal surface. The presence of such a silane monolayer on the metal surface decreased the water adsorption on the surface and inhibited the hydration of the metal oxide film. Moreover, formation of a negatively charged siloxane film improved the corrosion resistance while the positively charged layer enhanced the metal dissolution.

## 4. Silane Coatings for Corrosion Resistance of Mild Steel

Silane coating, one of the possible treatments, is found to have several attractive properties (such as being non-toxic, environment-friendly, low cost and easy to apply) to improve the corrosion resistance of different metals and alloys, including mild steel.

**Figure 5:**
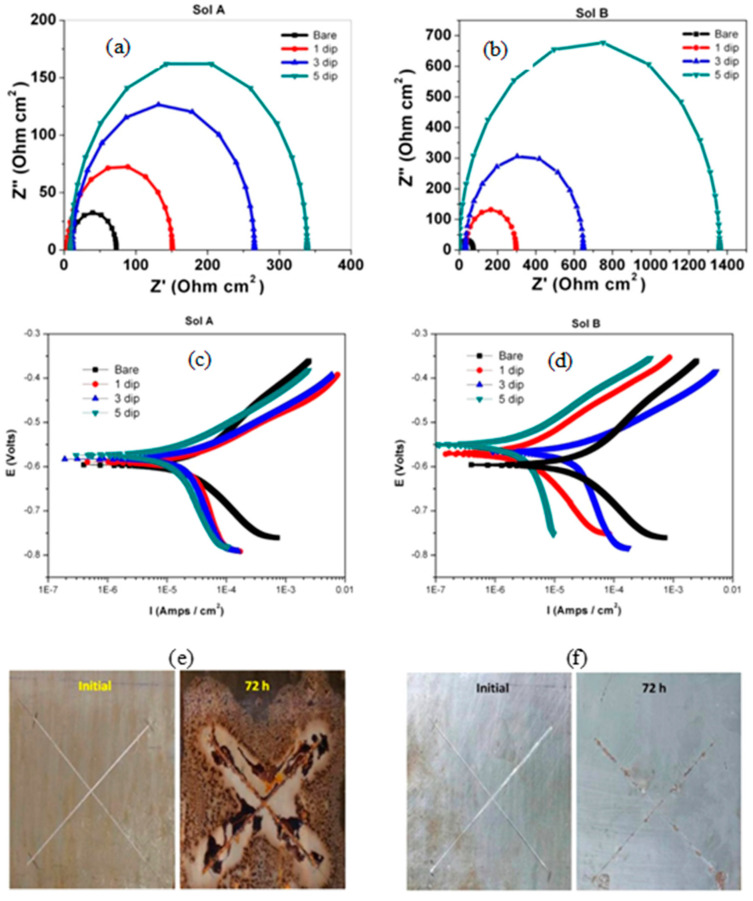
EIS plot (**a**,**b**) and Tafel plot (**c**,**d**) of sol-gel A and sol-gel B coating on mild steel in 3.5% NaCl solution. Salt spray testing of samples of sol-gel A and sol-gel B coatings on mild steel (**e**,**f**) [48].

Jeyaram et al. [48] synthesized silane coating of 3-glycidyloxypropyl trimethoxy silane (sol A) as the precursor and (3-aminopropyl) trimethoxysilane (sol B) as the cross-linking agent on mild steel. Electrochemical impedance spectroscopy (EIS) showed that dipping five times in silane solutions improved the corrosion resistance by three times due to sol A coating and 14 times due to sol B (Figure 5a,b, respectively). The extracted electrochemical parameters from EIS suggested that the coatings developed from sol B showed higher charge transfer resistance (R_ct_) and lower double-layer capacitance than the coatings from sol A. The R_ct_ of sol-B-coated mild steel increased from 65 Ω cm^2^ to 1370 Ω cm^2^, resulting in 98% protection efficiency compared to that of sol-A-coated with an efficiency of 80%. In addition, the reduction in double layer capacitance of sol B coating suggested that increasing the dipping time provides better corrosion protection. Such improvements were also reflected in polarization results, as the i_corr_ decreased from 282 µA/cm^2^ for uncoated steel to 87 and 62 µA/cm^2^, respectively, for the steel treated with sol A and sol B (i.e., five dips in each solution), as shown, respectively, in Figure 5c,d. In salt spray test for 72 h, blistering, red rust and the corrosion products are seen on the sol-A-coated mild steel, resulting in the peeling off of coatings. However, the coatings obtained from sol B showed no delamination and no red rust, suggesting that the coatings obtained from sol B are homogeneous, compact, dense, and pin-hole free and adhere to the mild steel’s surface (Figure 5e,f).

**Figure 6:**
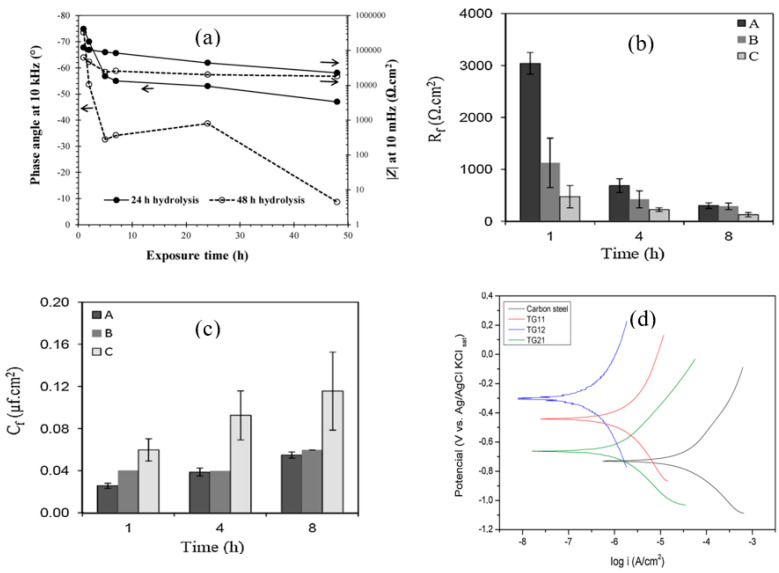
(**a**) The impedance magnitude (|Z|) at low frequency (10 mHz) and phase angle at high frequency (10 kHz) of silane coatings with hydrolysis times of 24 and 48 h [49]. (**b**,**c**) Evolution of silane film resistance (Rf) and capacitance (Cf) (A: pH 2.8, dipping time 120 s; B: pH 2.8, dipping time 60, pH 2.8, dipping time 30 s) within 8 h of exposure to 0.1 M NaCl solution [50]. (**d**) Potentiodynamic polarization (PDP) of the uncoated substrate and coatings with different molar ratios of 1:1,1:2 and 2:1 of TEOS and GPTMS [51].

Alibakhshi et al. [49] studied the effect of hydrolysis time and concentration of silane on the corrosion protection of mild steel; tetraethylorthosilicate (TEOS) and trimethoxymethylsilane (TMOMS) were used as silane coatings. EIS results revealed that the coating of the mixture of 50% silanes (TEOS/TMOMS: 50/50 *w*/*w*) hydrolyzed for 24 h provided greater corrosion resistance in 3.5% NaCl solution, due to the hydrophobic nature of the coating and better bonding with metal surface and formation of stable film as supported by SEM. The highest corrosion resistance was observed after 1 h of exposure, after which the resistance gradually dropped (as shown in Figure 6a), presumably due to the development of conductive pathways for the electrolyte diffusion through the coating. The phase angle values were more negative in the high-frequency regime for the silane coating with 24 h of hydrolysis compared to 48 h of hydrolysis, suggesting the better protection behavior of the former.

Other factors that influence the performance of silane coatings as a corrosion barrier are pH of hydrolysis and dipping time. Asadi et al. [50] investigated the effects of dipping duration (30, 60 and 120 s) in the silane solution and the pH of the solution (2.8–4.0) on the protection due to silane coating consisting of a mixture of tetraethoxysilane, methyltriethoxysilane and glycidyloxypropyltrimethoxysilane on mild steel. Electrochemical results showed that the coating developed by dipping the mild steel in the silane solution mixture for 120 s at pH 2.8 provided the most efficient protection with higher resistance and lower capacitance of the silane film, as shown in Figure 6b,c, which is attributed to the higher degree of cross-linking and homogeneity of the deposited coating.

Subramanian et al. [9] compared the corrosion inhibition due to silane coatings developed using two different silanes (i.e., γ-aminopropyltriethoxysilane (γ-APS) and bis-triethoxysilyl ethane (BTSE)). The silane films were developed on iron by dipping for 100 s and curing at 60 °C for 60 min in air. The films of the functional group silane (γ-APS) alone or the mixture of γ-APS and BTSE were found to have virtually no effect on the corrosion rate of iron. In contrast, the film of the non-functional silane (BTSE) alone developed at pH 4–6 decreased the corrosion rate by a factor of 15. In another related study [11], to investigate the influence of the molecular structure of silane monomers on the corrosion protection, organofunctional (γ-APS), bis-organofunctional (bis(trimethoxysilylpropyl)amine (BAS)) and bis-non-functional (BTSE) silanes were deposited on cold-rolled steel. Electrochemical tests in a relatively benign environment of 0.39 M Na_2_SO_4_ showed that the rise in the number of silanols, produced from the fully hydrolyzed silane and the absence of an amine group, would enhance the cross-linking reactions during the curing process. The greater the cross-linking density, the better is the corrosion resistance. Pepe et al. [52] studied the electrochemical corrosion of carbon steel coated with a hybrid organic–inorganic silica sol–gel. The coatings were developed by immersion of the samples in an organically modified silica sol made from hydrolysis and polycondensation of tetra-orthosilicate (TEOS) and methytriethoxisilane (MTES) by acidic catalysis. After exposure for 30 min at room temperature, the treated substrates were cured for 15 min at 400 °C and at the atmospheric pressure. The coatings thus developed showed an effective barrier against corrosive environment in the initial stages of immersion; however, the coating performance deteriorated significantly after 48 h of pre-immersion in corrosive media.

Alcantara-Garcia et al. [51] investigated the influence of different molar ratios of silanes on the chemical structure, physical properties and corrosion resistance of carbon steel. Three different molar ratios were used to prepare the organic–inorganic hybrid materials by hydrolysis and condensation of (3-glycidyloxypropyl) trimethoxysilane (GPTMS) and tetraethylorthosilane (TEOS) in an ethanol/water solution. Potentiodynamic polarization (PDP) results showed the E_corr_ to significantly shift to more noble potentials, in particular for the coating prepared with a 1:2 molar ratio of TEOS to GPTMS when compared with the uncoated substrate. On the basis of the corrosion current density, the coating with a molar ratio of 1:2 showed the lowest corrosion rate, followed by coatings of 2:1 and 1:1 ratios (Figure 6d) [51].

Chico et al. [53] investigated the effect of curing time on the barrier properties of two types of silane, 3-aminopropyltriethoxysilane and bi-3-triethoxysilylpropyl amine, applied on steel substrates, with and without a subsequent application of alkyd-based paint. For samples with silane treatment alone, a benign medium (0.5 M potassium sulphate) was used as electrolyte, whereas 0.6 M NaCl solution was used for the metal/silane/paint system. The electrochemical impedance spectroscopy (EIS) showed the barrier properties and corrosion protection to be closely related to the prior curing process and the extent of curing. The individual silanes (e.g., bis-(trimethoxysilylpropyl)amine (bis-amino silane) or bis-(triethoxysilylpropyl)tetrasulphide (bis-sulfur silane)) performed poorly on cold-rolled steel [54] in hot salt soak and GM scab tests, whereas a mixture of the two silanes (2% prehydrolyzed concentration each) with a mixing ratio of 9:1 for bis-amino and bis-sulfur could provide corrosion performance as good as that due to a zinc phosphate system.

Wang et al. [55] studied the influence of cleaning solution pH on the cold-rolled steel’s surface chemistry and the corrosion resistance of silane-coated steel. In this study, a part of the as-cleaned cold-rolled steel substrates was coated with 10wt% aqueous solution of bis-amino silane and vinyltriacetoxysilane (VTAS) in a weight ratio of 5:1. Another part of the substrates was coated with a silane-containing primer (80wt% epoxy resin, 10wt% bis-sulfur silane, 9wt% amino silane/VTAS mixture and tetraethoxysilane). PDP measurements showed that the i_corr_ decreased by half of an order of magnitude when the steel substrates were pre-treated with the cleaning solution at pH 9.5 prior to silane deposition, which was also supported by EIS data.

In a series of studies [56,57,58,59], one- and two-dimensional polymer films have been developed on iron surface by modification of a monolayer of either 11-Mercapto-1-undecanol (MUO), N,N-dimethylalkylamine or p-hydroxymethaylbenzene with different silanes. The coating procedures were carried out in deoxygenated atmospheres (i.e., under nitrogen atmosphere), and the contact time with silane solutions was varied from 30 min to many hours. Nozawa and Aramaki [56] prepared one- and two-dimensional polymer films by modification of an 11-Mercapto-1-undecanol (MUO) monolayer on iron with tetraethoxysilane (TES) for 30 min at 40 °C, octyltriethoxysilane (C_8_TES) for 30 min at 40 °C and/or octadecyltriethoxysilane (C_18_TES) for 10 h at 40 °C. The protective efficiencies of the coatings were determined by polarization and EIS tests in 0.5 M NaCl. Although, the “protective efficiency” of the one-dimensional polymer film developed by modification of the MUO monolayer with C_18_TES for 10 h was quite high (92%) in an aerated NaCl solution, the “protective efficiency” improved by over 93% due to the coating developed upon two-dimensional polymer films. The two-dimensional films were produced by modification of the MUO monolayer adsorbed on iron with TES and (C_8_TES) or (C_18_TES). The films were composed of a two-dimensional polymer of the thiolate attached on the surface and interconnected with siloxane bonds and small amounts of iron oxide/hydroxide and water. Aramaki [57] reported that the “protective efficiency” of ultrathin two-dimensional film of BTSE plus C8TES-modified MOU layer was not very high (91.6%). A considerably high protective efficiency (98.1%) was achieved when the MUO layer was modified with BTSE followed by C_18_TES treatment for 48 h. This film also protected iron from atmospheric corrosion for 60 days [57]. Tsuji et al. [58] modified N,N-dimethylalkylamine monolayer with alkylchlorosilanes for developing protective films of two-dimensional polymers against iron corrosion in 0.5 M NaCl solution. The prolonged modification treatment (more than 24 h) of monolayer with a solution of octadecyltrichlorosilane (C_18_TCS) was applied to provide a regular arrangement of large C_18_ alkyl tails within the film. The calculated “protective efficiency” from EIS and polarization tests were 92.9% and 93.1% for the layer modified with 1,2- bis(trichlorosilyl)ethane (BTCSE) plus C18TCS. The “protective efficiencies” were far lower than those obtained for the MUO layer modified with BTSE and C_18_TES, which has been described in reference [57]. Shimura et al. [59] attempted to introduce an additional modification of ultrathin two-dimensional polymer film of p-hydroxymethaylbenzene self-assembled monolayer. The monolayer was modified with BTSE and alkyltriethoxysilane C_n_H_2n+1_Si(OC_2_H_5_)_3_ (C_n_TES, *n* = 8 or 18). The protective ability of the modified coating against iron corrosion in 0.5 M NaCl solution after pre-immersion for 1.5 to 72 h was tested by polarization measurement. They found that the protective ability for the self-assembled monolayer modified twice with BTSE (for 2 h at 40 °C) followed by C8TES (for 2 h at 40 °C) did not improve markedly in comparison to that of the film without additional modification with BTSE, whereas the additional modification of the polymer films of BTSE plus C8TES (for 2 h at 40 °C) and BTSE plus C18TES (for 91 h at 40 °C) with C8TES for 2 h improved the barrier properties against corrosion. The enhancement was due to improvement of the alkyl tail arrangement and additional interconnection in polymer films.

## 5. Approaches for Improving Silane Coatings

### 5.1. Metal Oxide and Metal Oxide-Impreganated Silane Coatings

Metal oxides such as SiO_2_, ZrO_2_, Al_2_O_3_, TiO_2_ and CeO_2_ are resistant to corrosion [60]. Several studies have investigated the effect of impregnation of such metal oxides into silane coatings on the corrosion resistance of metals and alloys. This section reviews the enhancement in corrosion protection of mild steel due to silane coatings impregnated with such metal oxide.

Vivar Mora et al. [61] prepared sol–gel incorporated non-functionalized and functionalized nanoparticles of silica (SiO_2_) for corrosion resistance of mild steel. Three different molar ratios of tetraethylorthosilicate (TEOS) and 3-glycidoxypropyltrimethoxysilane (GPTMS) were used to develop a coating with thickness greater than a few microns to accommodate the added nanoparticles. Salt spray test results showed that the addition of non-functionalized silica nanoparticles improved corrosion resistance, though corrosion is evident after 72 h. On the other hand, the corrosion resistance improved due to incorporating GPTMS-functionalized silica nanoparticles, showing very little corrosion even after 72 h. Li et al. [62] coated Q235 carbon steel with a two-layer coating composed of a primary underlying layer impregnated with silica and titania powder as filler and pigment materials and a translucent topcoat layer containing a colloidal silica sol–gel matrix cross-linked by methyltrimethoxysilane (MTMS). They noticed no visible signs of corrosion for the first 28 days of the salt spray test. However, after 35 days, the corrosion spots started to be visible. EIS tests in 5% NaCl solution revealed that the low-frequency impedance value |Z|_0.01Hz_ is in the range of 10^8^–10^9^ Ω·cm^2^, which is relatively high compared to other coating systems.

ZrO_2_ is another metal oxide that finds a variety of applications, including coatings for improving the corrosion resistance of metals and alloys [63,64,65,66,67]. Incorporating ZrO_2_ nanoparticles into sol–gel coatings improves coatings’ mechanical properties [68]. Claire et al. [68] reported a bi-layer sol–gel coating system, with the outer layer containing zirconia nanoparticles, to improve the corrosion and abrasion properties of carbon steel. Kiruthika et al. [69] investigated a hybrid sol–gel based nanocomposite coating composed of GPTMS with zirconium-n-propoxide on mild steel. The coating enhanced the abrasion resistance (to withstand up to 500 cycles), as well as corrosion resistance.

**Figure 7:**
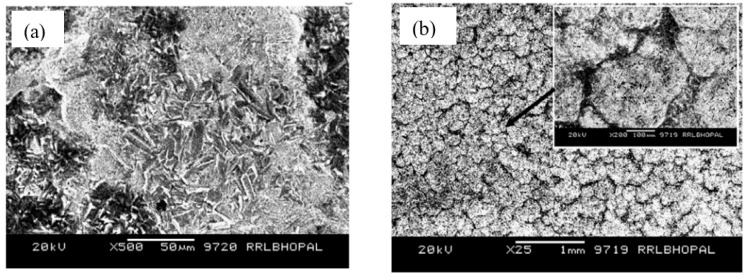

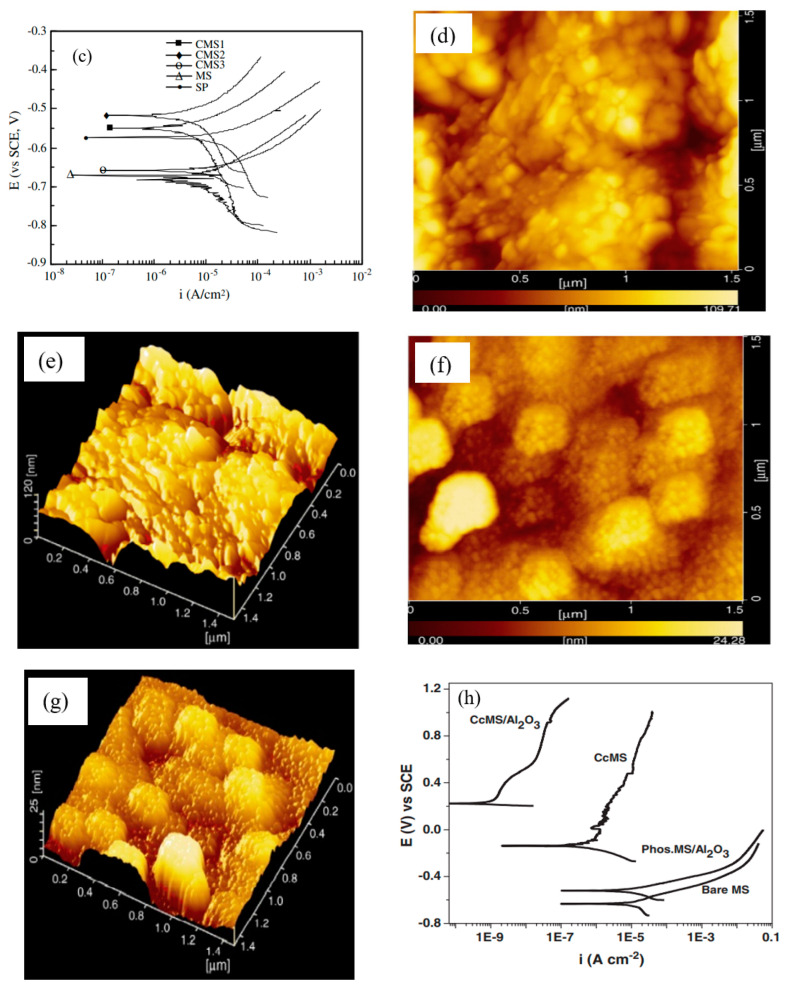
Surface morphology of the sol–gel-coated specimens sintered at (**a**) 400 °C and (**b**) 500 °C [71]. (**c**) PDP plots for the mild steel (MS), the surface pre-treated mild steel (SP) and the sol–gel-coated specimens sintered at 300 °C (CMS1), 400 °C (CMS2) and 500 °C (CMS3) in 3.5% NaCl solution at room temperature [71]. AFM micrographs of coated surface: (**d**,**e**) conversion-coated mild steel (CcMS) and (**f**,**g**) alumina-sol-coated steel (Al2O3/CcMS). (**h**) PDP plots recorded at a scan rate of 0.5 mVs^−1^ in 3.5 wt% NaCl [72].

Among the various oxides employed in coating applications, alumina is widely used as an additive for improving the wear and corrosion resistance of coatings, since it is inexpensive, chemically inert and thermally highly stable [70]. Ruhi et al. [71] developed sol–gel alumina coatings on the zinc-phosphated mild steel, followed by sintering at 300, 400 and 500 °C. A high magnification SEM micrograph showed a rough but crack-free coating developed at 300 and 400 °C (Figure 7a for the coating developed at 400 °C). However, numerous cracks were observed in the coating sintered at 500 °C (Figure 7b) due to loss of plasticity as a result of the elimination of the organic part, as detected by FTIR analysis of the coating. As a result, PDP tests (Figure 7c) revealed that the coating sintered at 500 °C possesses the lowest corrosion resistance. However, the sol–gel coated specimen sintered at 400 °C showed a minimum corrosion current density i_corr_. Tiwari et al. [72] prepared a conversion coating by coating the mild steel with silica sol and dipping the dried silica sol-coated sample in aluminum oxy-hydroxide sol. Then, the coated sample was heated at 500 °C for 2 h and cleaned with ethanol after cooling. For comparison, the mild steel coated with conversion sol–gel is recoated by dipping it in alumina sol and drying at room temperature for 30 min, followed by heating at 250 °C for 15 min. This coating process is repeated five times, followed by heating at 500 °C for 1 h. AFM micrographs in Figure 7d,e showed that the average roughness (Ra), measured diagonally from a 40 μm × 40 μm area scan, is nearly 110 nm and 25 nm for conversion-coated mild steel (CcMS) and alumina-sol-coated steel (Al_2_O_3_/CcMS), respectively. The AFM micrographs also revealed that a layered structure sol–gel coating is orientated in different directions with circular nanoparticles of less than 20 nm diameters (Figure 7d,e). The cluster of particles (≈1 μm size) on the CcMS suggests continuous growth in all directions. In contrast, a uniform alumina sol coating is seen on the steel surface. Small dot-like features can be observed as approximately 300–350 nm diameter agglomerates (Figure 7f,g). Figure 7h shows the polarization behavior of CcMS, CcMS/Al_2_O_3_ and bare. The alumina coating on pre-phosphated mild steel (Phos.MSAl_2_O_3_) was also shown for comparison. Corrosion current densities (i_corr_) for CcMS and CcMS/Al_2_O_3_ are reduced by about two and five orders of magnitude, respectively, compared to bare MS substrate.

Due to its excellent chemical stability, heat resistance and low electron conductivity, TiO_2_ can be used as an excellent anti-corrosion material. It was found that applying sol–gel TiO_2_ coating or sol–gel SiO_2_ coating improved the corrosion resistance of mild steel in NaCl solution [73]. In contrast, the sol–gel film containing TiO_2_-SiO_2_ together provides the lowest corrosion protection, even lower than the bare mild steel. However, Krishna et al. [74] prepared the oxidation-resistant TiO_2_-SiO_2_ thin film coating on mild steel via the sol–gel process. They reported that a single-layer coating is sufficient to protect the substrate from oxidation with an optimized surface roughness.

**Figure 8:**
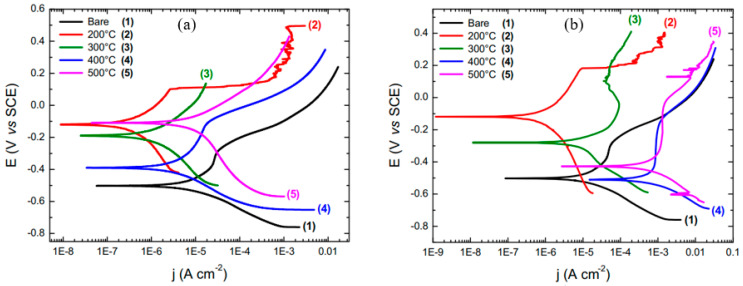
PDP curves in aqueous solution of NaCl 3.5% for electrodes coated with: (**a**) ceric ammonium nitrate and (**b**) cerium chloride, calcined at different temperatures and with heating rate of 5 °C/min [76].

CeO_2_, a rare earth oxide that has been extensively studied, has attracted the attention in thermocatalysis, electrocatalysis and photocatalysis applications [75]. For the corrosion protection of carbon steel, Carvalho et al. [76] studied the influence of the deposition of cerium oxide films produced from the incorporation of ceric ammonium nitrate and cerium chloride as metal precursors in sol–gel. The effect of using different temperatures and heating rates during thermal calcination was also studied. PDP results showed that cerium deposition enhanced the corrosion resistance of carbon steel, and the E_corr_ shifted to the noble direction. The corrosion current densities (i_corr_) decreased when a 5 °C/min heating rate was used during the prior thermal calcination. The changes are more obvious when the calcination temperatures was 200 °C (Figure 8a,b). They concluded that the sol–gel coatings with ceric ammonium nitrate as a cerium precursor developed at low calcination temperatures and high heating rates are promising as barrier protection for carbon steel and other metallic surfaces.

**Figure 9:**
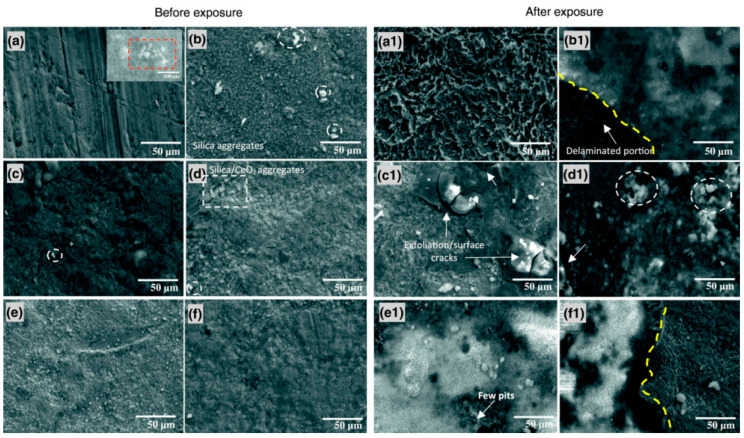
SEM micrographs of the coated mild steel before (**a**–**f**) and after (**a1**–**f1**) 14 days of exposure to aerated 0.6 wt% NaCl plus 0.6 wt% (NH_4_)_2_SO_4_ solution: (**a**) the metal etched surface without and (**b**) with the silane coating, the coating modified with (**c**) 0.25, (**d**) 0.50, (**e**) 1 and (**f**) 2 wt% CeO_2_. (Inset at (**a**): CeO_2_ nanoparticle aggregates (25 nm particle size distribution)) [77].

The epoxy–silica hybrid film of (3-glycidoxypropyl)-1,1,3,3-tetramethyldisiloxane and tetraethyl orthosilicate is synthesized for mild steel protection in a solution of 0.6 wt% NaCl and 0.6 wt% (NH_4_)_2_SO_4_ [77]. CeO_2_ pigments (0.25–2 wt%) are introduced as a filler to improve the barrier protection. EIS results revealed that using CeO_2_ pigments improved protection against the corrosion of mild steel compared to the unmodified silane coating, suggesting the formation of stable oxide/hydroxides of Ce within the coating network. Figure 9 compared SEM micrographs of coated mild steel before and after immersion in an aerated solution of 0.6 wt% NaCl plus 0.6 wt% (NH_4_)_2_SO_4_ for 14 days. White patches of silica and/or silica/CeO_2_ aggregates after the thermal curing were observed to be intact on the coated steel (Figure 9a–f). After the two-week immersion, some portions of the coatings are still intact, though micro-cracks and exfoliation were observed in the coating (Figure 9a1–f1). The improvement in corrosion resistance of the coated steel is due to the anticorrosive properties of CeO_2_ and the pigment enforcing interconnectivity.

### 5.2. Silane Incorporated with Plant Extracts

The hazards associated with the use of common synthetic corrosion inhibitors such as chromate triggered the need to develop nontoxic, inexpensive and environment-friendly approaches, such as the use of silane-based coatings and green inhibitors [78]. For example, the effect of incorporating caffeine (which is extracted from tea leaves) into the hybrid silane coating of TEOS and APTES to improve the corrosion resistance of mild steel in 3.5 wt% NaCl solution was investigated by Hamidon and Hussin [79]. They found that caffeine-doped silane coating improved the corrosion resistance of steel in NaCl solution, with 100 ppm caffeine providing the maximum improvement.

Ishak et al. [80] studied the influence of doping the TEOS-APTES sol–gel with curcumin in mild steel corrosion in a 0.5M HCl solution. PDP tests showed i_corr_ to decrease with the increase in the concentration of the curcumin in the silane film; the optimum concentration was 100 ppm. Hamidon et al. [81] doped the same sol–gel system (TEOS-APTES) with different concentrations of aqueous crude extract of span tea leaves (0, 25, 50, 75 and 100 ppm). Doping the sol–gel with span tea extract improved the corrosion resistance of steel in NaCl solution, and the optimum concentration of span tea extract was 75 ppm, as suggested by PDP and EIS tests. Ghuzali et al. [82] incorporated the extracts of Clitoria ternatea (using water and ethanol as solvents) into the hybrid sol–gel of GPTMS, using TEOS as the precursor to enhance the corrosion resistance of mild steel in 0.5M HCl solution. Electrochemical impedance spectroscopy data suggested that at 75 ppm of the Clitoria ternatea extract in the sol–gel coating, the inhibition efficiencies were 89.6% and 85.6% for the coating; these green inhibitors were extracted using ethanol and water, respectively.

### 5.3. Silane Coatings Impregnated with Graphene Oxide

Graphene is an attractive 2D material with remarkable physical and chemical properties for diverse applications [83]. The tiny pores in the hexagonal lattice structure of graphene (i.e., 0.064 nm) are impermeable to fluid molecules; hence, the use of graphene coating is a promising tactic for attaining durable corrosion resistance of metals and alloys [83].

**Figure 10:**
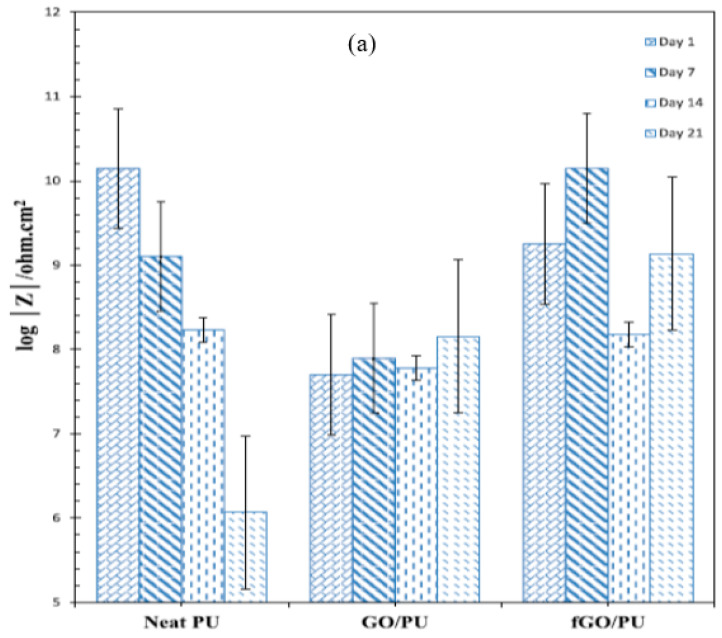

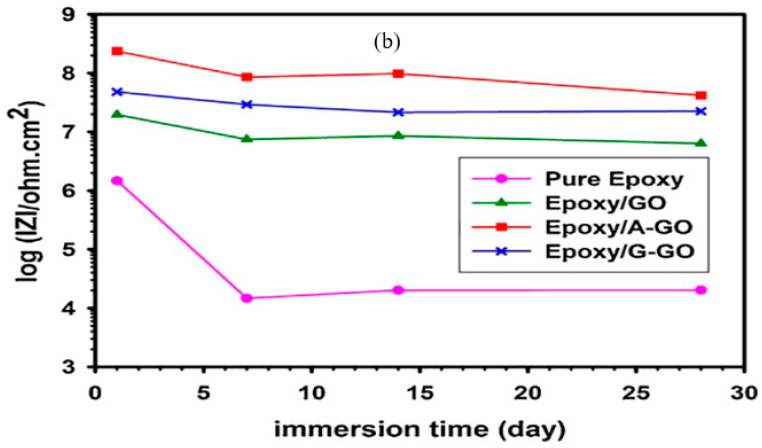
(**a**) Impedance (|Z|) at the lowest frequency (10 mHz) extracted from Bode plots for neat PU, GO/PU and fGO/PU after immersion for different durations in 3.5 wt% NaCl [84] and (**b**) evolution of |Z| for pure epoxy and GO-nanocomposite-impregnated epoxy coatings during immersion in 3.5 wt% NaCl solution for 28 days [85].

Haghdadeh et al. [84] functionalized the graphene oxide (GO) nanosheets with (3-glycidyloxypropyl) trimethoxysilane to be introduced into the polyurethane (PU) matrix for improving the corrosion resistance and mechanical properties of the composite coating on metals. The corrosion protection properties of the coatings were investigated using salt spray and EIS tests. In the salt spray test, blisters and corrosion spots emerged on both the GO/PU and neat PU coatings after 300 h and 600 h, respectively, that increased with the duration of salt spray. Thus, incorporating the neat GO into the PU matrix was found to be deleterious for corrosion resistance, which was attributed to the presence of many polar groups on the GO sheets and their ability to increase the hydrophilic nature of PU, thereby increasing susceptibility to corrosion. This mechanistic explanation was duly supported by the fact that when PU was added with suitably functionalized GO, i.e., fGO (instead of plain GO), corrosion resistance due to fGO/PU coating improved, which is attributed to the hydrophobic nature of fGO. With fGO/PU coating, considerably fewer corrosion spots and blisters were observed even after 900 h of salt spray. Figure 10a shows the evolution of impedance (|Z|) in the lowest frequency regime (10 mHz), i.e., representation of corrosion resistance, for the neat PU, GO/PU and fGO/PU during immersion for different durations in 3.5 wt% NaCl solution. Corrosion resistance decreased rapidly for the neat PU-coating (Figure 10a). Consistent with the salt-spray test results, the corrosion resistance of GO-containing PU coating (GO/PU) was inferior to the plain PU coating, since GO is known to suffer agglomeration that creates defects in the coating. However, suitably functionalization of GO to develop fGO/PU coating provided considerably improved and durable corrosion resistance, as seen in Figure 10a [84].

Pourhashem et al. [85] modified 3-aminopropyl triethoxysilane (APTES) and 3-glysidyloxypropyl trimethoxysilane (GPTMS) silanes to create amine and epoxy end-groups and then used the modified silanes to functionalize graphene oxide (GO). The composite coating was applied onto steel. Corrosion resistance of bare, epoxy-coated and nanocomposite-epoxy-coated steel in 3.5 wt% NaCl solution was evaluated. OCP of coatings was evaluated to be positive in the order: epoxy < epoxy/GO < epoxy/G-GO < epoxy/A-GO, i.e., the addition of nanofiller improved the corrosion resistance, which is also corroborated by the corresponding impedance (|Z|) data (Figure 10b). The corrosion resistance decreased with increasing immersion time; however, the decrease is considerably less pronounced for epoxy coatings modified with GO and its derivatives. As seen in Figure 10b, the impedance (|Z|) of steel coated with unmodified (pure) epoxy decreased rapidly from ~10^6^ to ~10^4^ Ω cm^2^ within 7 days of immersion, before stabilizing. On the other hand, impedance (|Z|) stays reasonably stable in the case of epoxy coatings modified with GO and its derivatives, suggesting their ability to overcome the deleterious effect of porosity and defects of the nanocomposite coating. In another study, Pourhashem et al. [86] investigated epoxy coatings containing graphene oxide (GO) and amino-silane modified GO (A-GO), with various weight fractions (0.05, 0.1, 0.3 and 0.5 wt%), on mild steel 3.5% NaCl solution. They found the corrosion resistance due to the epoxy/A-GO coating to be significantly higher. The optimum GO content was found to be 0.1wt%; at greater contents, GO nanosheets started to suffer agglomeration.

Graphene oxide (GO)-based nano-impregnation containing silane composite coatings also provides superior corrosion resistance to mild steel in concrete solution containing chloride [87]. In the alkaline environment of concrete (pH > 10), mild steel can form a protective passivation layer; however, the passive layer can be disrupted by chloride ions, resulting in rapid corrosion of steel at the locations of disruption [88,89]. Geng et al. [87] investigated GO-modified silane coating developed on steel reinforcement bars in a simulated concrete pore solution containing 3.5% NaCl, and reported the coating to provide excellent corrosion resistance, with a maximum protection efficiency of ~99%. Sharma et al. [90] coated the mild steel rebars with an epoxy coating using reduced graphene oxide (rGO) and graphene oxide (GO) along with carbon nanotubes (CNTs) and silane agents for improving corrosion resistance in concrete. They found that the addition of rGO and GO with CNTs to pure epoxy produces a dense, impermeable and durable coating on steel, as compared to pure epoxy layer. The epoxy alone coating impedes corrosion initiation for 8 days, whereas rGO/CNT-epoxy delays it by up to 50 days. The long-term corrosion protection performance is superior for GO/CNT-epoxy coating, as compared to rGO/CNT-epoxy coating.

Table 1 summarizes the different approaches for improving silane coatings and the main findings, as reported in the literature.

## 6. Silane Coatings for Mitigation of MIC and Biofouling of Mild Steel

Biocorrosion or microbiologically influenced corrosion MIC can be defined as an electrochemical process where the microorganisms participate in initiating, facilitating or accelerating the corrosion reaction without altering its electrochemical nature [91]. MIC becomes a frequent risk factor for accidents such as fluid flooding, leakage and rupture when the equipment in different industries is used for a long time [92].

Besides their use for general corrosion protection, silanes and silanes impregnated with other environment-friendly and non-hazardous materials can mitigate microbiologically influenced corrosion (MIC) [93].

**Figure 11:**
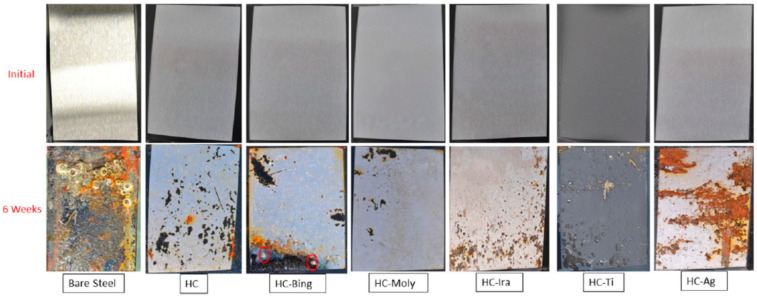
Field trial samples of all coating matrices compared with bare/uncoated steel after 6 weeks of exposure to seawater in the Eastern Province of Saudi Arabia, which has minimal flow and is known for its marine macrofouling activities and high salinity [94].

Suleiman et al. [94] synthesized a hybrid sol–gel material (HC) from allyl precursors and functionalized it into hybrid polymeric material incorporated with various organic and inorganic antibacterial agents, i.e., 1,1-dimethylbiguanide hydrochloride (HC-Bing), Irgarol (HC-Ira), MOLY-white 101 (HC-Moly), Ti nanoparticles (HC-Ti) or silver nanoparticle dispersion (HC-Ag). EIS results for the coated samples exposed to plain 3.5% NaCl solution showed that HC, HC-Ira and HC-Moly had considerably higher corrosion resistance, which was supported by i_corr_ data in PDP tests. In contrast, it was HC-Bing and HC-Ti that showed greater resistance to biofouling. As seen in Figure 11, only two hard-shell barnacles were found to become attached to HC-Bing during 6 months of a marine biofouling test, whereas the bare steel substrate suffered heavy biofouling and corrosion. In comparison, no barnacles, polychaetes or deposits were observed on the surfaces of other coatings.

**Figure 12:**
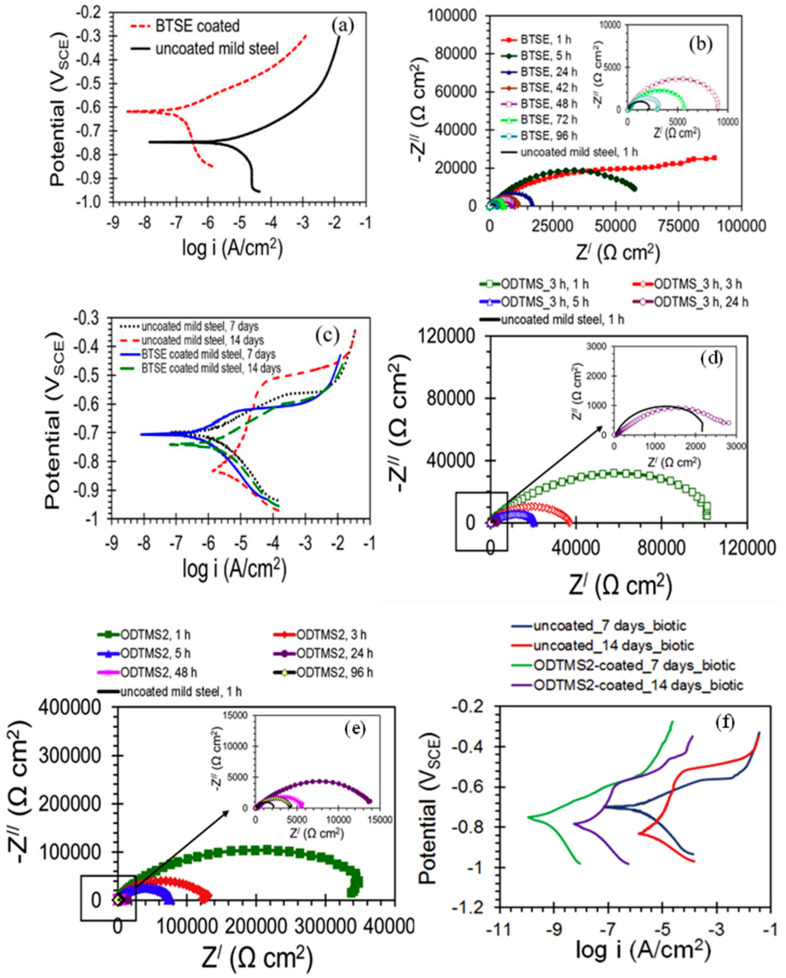
(**a**) PDP plots of the BTSE-coated and uncoated mild steel pre-immersed for 1 h in 0.6 M NaCl solution, (**b**) Nyquist plots for the BTSE-coated specimens at different durations of immersion and the uncoated mild steel specimens at 1 h of immersion in 0.6 M NaCl, (**c**) PDP plots of the BTSE-coated and uncoated specimens after different exposure times in the marine environment with SRB [95], (**d**) Nyquist plots for uncoated and ODTMS-coated mild steel pre-immersed in 3.5% NaCl solution for 1, 3, 5 and 24 h, (**e**) Nyquist plots for the two-step coated (ODTMS2) and uncoated mild steel pre-immersed in 3.5% NaCl solution up to 96 h and (**f**) PDP curves of ODTMS2-coated and uncoated mild steel specimens after exposures for 7 and 14 days to marine environment inoculated with SRB [96].

Al-Saadi et al. [95] studied the influence of Bis[triethoxysilyl]ethane (BTSE) silane coating on the corrosion resistance of mild steel in an aerobic chloride medium and an anaerobic microbial medium containing sulphate-reducing bacteria (SRB). BTSE coating significantly enhanced the corrosion resistance of mild steel in 0.6M NaCl; the i_corr_ of the uncoated steel was at least 25 times higher than that of the steel coated with BTSE (Figure 12a). EIS results in Figure 12b showed that the impedance of the BTSE-coated steel was 45 times higher than that of the uncoated mild steel. In the presence of SRB, BTSE coating provided little protection to the mild steel substrates as suggested by the more negative E_corr_ and the significant increase in i_corr_ after 14 days of exposure to marine environment inoculated with SRB (Figure 12c). They reported that the limited improvement provided by BTSE coating in the presence of SRB is due to the absence of any antimicrobial functional group in this silane.

Al-Saadi and Raman [96] developed octadecyltrimethoxysilane (ODTMS) coatings upon single- and two-step treatments for enhancing the corrosion resistance of mild steel in marine environments with and without SRB. The low-frequency impedance |Z| for the ODTMS-coated steel developed upon one-step treatment was ~100 kΩ cm^2^, which is ∼50 times higher than that for the uncoated steel (~2 kΩ cm^2^) after 1 h of exposure to NaCl solution, as shown in Figure 12d. For the coating deposited by two-step treatment (i.e., ODTMS2), the impedance |Z| increased by more than two orders of magnitude compared to that for the uncoated specimen. After 24 h of pre-immersion in NaCl, though the corrosion resistance of coated steel decreased by about 20 times as compared to 1 h pre-immersed coated steel, the two-step coatings still provided significant protection, as shown in Figure 12e. In the marine environment with SRB, PDP results (Figure 12f) for the uncoated mild steel revealed that the E_corr_ shifted significantly towards the cathodic region (the potential moved ~128 mV more negative during the period of exposures of 7–14 days), whereas, for the mild steel coated with ODTMS2, the potential shifted only by ~35 mV towards the positive direction, suggesting considerably less susceptibility to anodic dissolution compared to the uncoated steel. The i_corr_ values of the uncoated steel were significantly higher than those for ODTMS2-coated specimens, for exposures of 7–14 days, in the marine environment with SRB. The i_corr_ values for uncoated steel were more than three orders of magnitude higher (for 14 days) and two-and-half orders of magnitude higher (for 7 days) than those for ODTMS2-coated steel. They reported that the improvement in corrosion resistance is attributed to the formation of the highly crystalline, densely packed, hydrophobic and orientated film due to the long aliphatic chain silane. [96].

**Figure 13:**
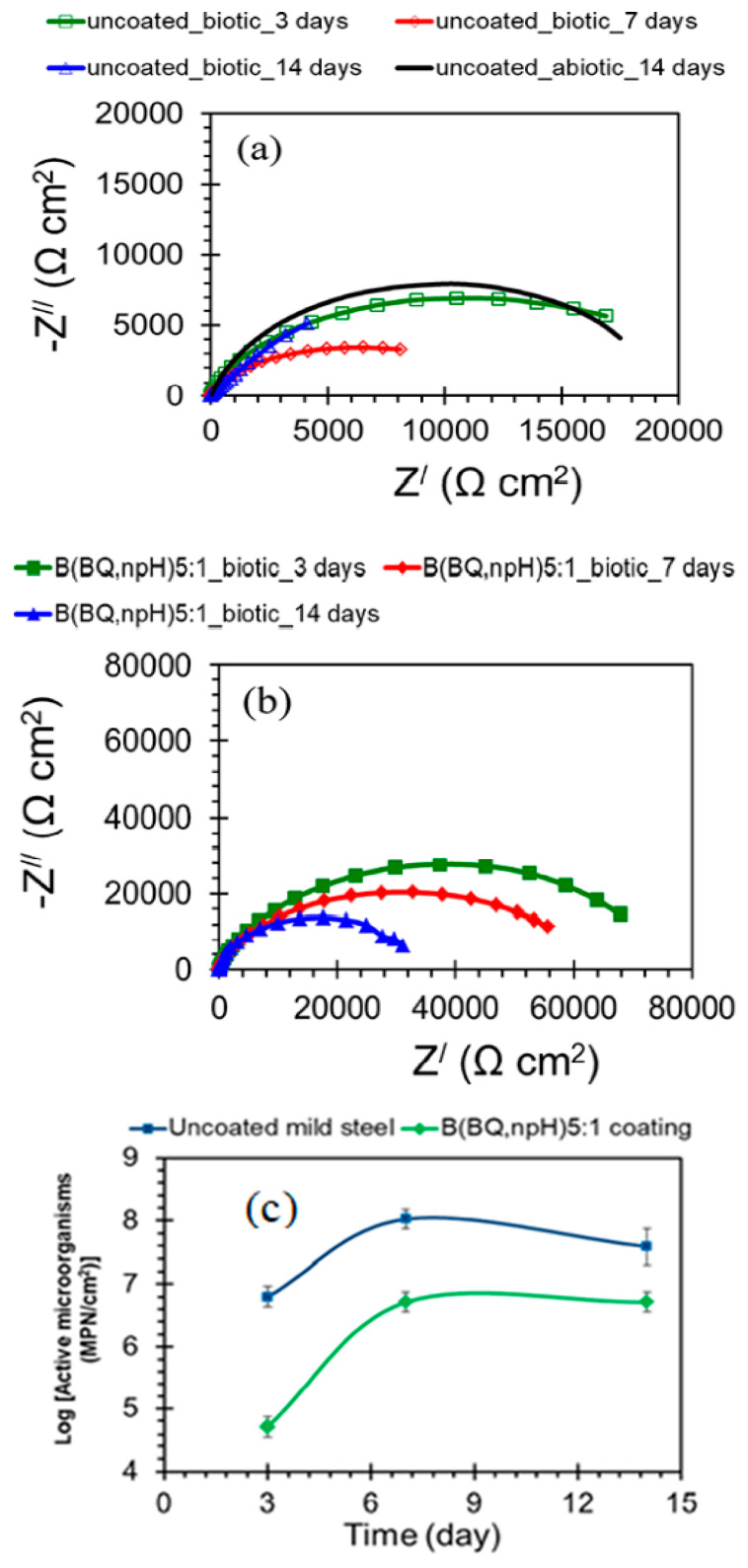
(**a**,**b**) Nyquist plots of uncoated and silane-coated mild steel after exposure times of 3, 7 and 14 days in a marine environment inoculated with SRB. (**c**) SRB population on uncoated and two-step silane-coated mild steel surfaces with respect to the time of exposure to the marine environment with SRB [93].

Quaternary ammonium silanes (QAS) have shown antimicrobial and antibacterial activity. The antibacterial materials can attack the cell membrane and turn it semipermeable, causing leaking of the cell contents [91]. The electrostatic contact between the quaternary ammonium compounds and the negatively charged surfaces of bacteria facilitates interaction and penetration by the alkyl chain through the bacteria’s membrane, causing the bacterial cell’s dissolution and death [92]. Al-Saadi et al. [93] studied the inhibition action of the two-step coating of a non-functional silane, BTSE, followed by another top coating with a silane mixture of bis-[trimethoxysilylpropyl]amine (bis-amino silane) and 3-[trimethoxysilyl]-propyldimethyloctadecyl ammonium chloride (QAS) in a mixing ratio of 5:1 (*v*/*v*) for resistance of mild steel to MIC in a chloride solution with SRB. Impedance results in Figure 13a show that the corrosion resistance of the uncoated steel in the marine solution with SRB decreased with the increase in the duration of exposure, i.e., from 17.8 kΩ cm^2^ at 3 days to 8 kΩ cm^2^ at 7 days, and after 14 days, the impedance dropped down to 4 kΩ cm^2^. However, impedances (|Z|) of steel with the two-step silane coating incorporated with the quaternary ammonium silane were significantly higher than those for uncoated specimens, clearly evidencing the two-step silane treatment to have noticeably improved the corrosion resistance of the coated mild steel in the marine environment with SRB, as seen in Figure 13b. The most probable number (MPN) technique showed that the active SRB cells on the uncoated surface pre-exposed for 3 days to the marine environment with SRB were about 6 × 10^6^ MPN/cm^2^ (Figure 13c). The number of active cells increased by more than 16 times after 7 days and remained higher by seven times after 14 days of pre-exposure. However, for silane-coated steel samples, the number of active cells adhering to the coated surfaces was much lower than those attached to the uncoated steel samples.

Though there is some reported literature on the role of silane coatings for mitigation of MIC, this is a vastly unexplored research topic, even though the topic may have huge industrial relevance.

## 7. Conclusions

Due to the health risk concerns of using chromate conversion coatings for metals and alloys, silane coatings have gained significant attention as alternative surface pre-treatments for adhesion promotion and corrosion resistance enhancement. This review discussed the recent work on silane coatings for mild steel against corrosion and microbiologically influenced corrosion (MIC). The salient features of the review are:

1- The hydrolysis and condensation of silanes are among the critical factors influencing the robustness of the coatings developed on the metal surface. Most of the silanes are subjected to acid-catalyzed hydrolysis, where a high rate of hydrolysis with slow gelation is preferred. On the other hand, in the base catalyzed reaction, the rate of condensation reaction is higher with fast gelation.
2- Sufficient silanol groups generated from hydrolysis are required that facilitate the subsequent condensation/bonding between silanol groups (-SiOH) and metal surface hydroxyls (MeOH).
3- Though different studies showed the ability of silane to improve the corrosion resistance of mild steel, the durability of the developed silane coatings can be improved by incorporation of functionalized and unfunctionalized metal oxides, plant extracts and 2D materials into the coatings.
4- In the corrosive environments of chloride with the presence of sulphate-reducing bacteria, the non-functional silanes provided little improvement due to the absence of any antimicrobial functional group in such silanes. However, the long aliphatic chain silane (e.g., octadecyltrimethoxysilane (ODTMS) and quaternary ammonium silane (QAS)) improved the corrosion resistance in the microbial environment due to the formation of hydrophobic surface in case of ODTMS coating or because of the antibacterial characteristics of QAS.

## Figures and Tables

**Table 1:** Different approaches for improving silane coatings and the main findings, as reported in the literature.

Silane(s) Used	Coating Preparation	Main Findings	Ref.
✓ Tetraethylorthosilicate (TEOS) ✓ 3-GPTMS (3-glycidoxypropyltrimethoxysilane) (GPTMS)	➢ Before the coating application, silica nanoparticles (SiO_2_) were impregnated into the optimized mixture of TEOS/GPTMS with a molar ratio of 1:1.8. ➢ In a second procedure, the silica nanoparticles were surface-treated with GPTMS for compatibility/bonding with the coating matrix and to prevent/reduce agglomeration of nanoparticles within the coating formulation. ➢ Coatings were manually applied on cleaned steel, followed by drying and curing at 90 °C for 2 h.	❖ Electrochemical tests showed that the silane coatings with and without silica nanoparticles had corrosion potentials higher than that of the uncoated metal, in 3.5% NaCl. ❖ Among all types of coatings, the sample coated with silane impregnated with non-functionalised silica nanoparticles had slightly higher potential, suggesting that the incorporation of non-treated silica nanoparticles produced the crack-free and less porous coated surface. ❖ After 24 h of exposure, the potential for coating with and without non-functionalised silica continued to drop, suggesting increased corrosion susceptibility, possibly due to water uptake in the coating. ❖ EIS results also revealed an increase in pore resistance, suggesting lesser ion-conducting paths in the coatings.	[61]
✓ Methyltrimethoxysilane (MTMS), 99%	➢ Liquid dispersion for undercoat: A slurry mixture of 2120 g of colloidal SiO_2_ suspension, 670 g of silica whisker powder and 500 g of TiO_2_ pigment powder was subjected to dispersion at 500 RPM/min for 30 min, followed by pH adjusting with 5% HCl and addition of MTMS and agitation at 300 RPM/min for 8 h at room temperature. ➢ Liquid dispersion for topcoat: 200 g of colloidal SiO_2_ suspension was mixed with 40 mL of distilled water in a high-speed dispersing machine for 10 min (pH adjusted to 3.0). Then, 120 g of MTMS was added, and the reaction was allowed for 7 h at room temperature under mixing at 300 RPM/min. ➢ Steel coupons were sandblasted by quartz sand, washed and rinsed using an ethanol/distilled water mixture with ultrasonication. Coating employing the undercoat and topcoat procedures as described above (with 5 min of drying between the two) was developed by pressurized spray process. Then, the coating was cured at 170 °C for 30 min, followed by cooling to room temperature in an ambient environment.	❖ SEM imaging showed that the coating thus developed was uniform, with little visible defects/micro-cracks. Closely packed SiO_2_ nanoparticles became embedded in the top layer. Features of the underlying layer included a random distribution of filler materials forming a rougher surface. ❖ Salt spray test results revealed no visible corrosion sites during 28 days; however, occasional spots started to appear after 35 days and increased in size with time, but the rest of the coated surface remained intact up to 56 days. ❖ The impedance value at low-frequency |Z|_0.01Hz_ fell in the range of 10^8^–10^9^ Ω·cm^2^, which is relatively high compared to other coating systems. In the phase angle diagram, the low-frequency time constant for the two-layer coating is highly unlikely to correspond to the coating/metal interface since there was no indication of any large mass transport of corrosion products between the metallic surface and the corrosive environment. The Bode plots of the two-layer coating did not show a significant drop in impedance with increasing immersion time, which is indicative of the durable barrier effect that enables long-term performance.	[62]
✓ Glycidoxypropyltrimethoxysilane (GPTMS)	➢ The primer coating (S0): GPTMS (5.94 g, 0.0251 mol) and aluminum isopropoxide AIP (3.6 g, 0.0176 mol) in a molar ratio of 1.7 were mixed in isopropanol 11.25 mL (0.1479 mol). Cerium nitrate hexahydrate (1.00 g, 0.0023 mol) was dissolved in distilled water (25.8 g, 1.43 mol) and then mixed with the previous mixture. The sol was prepared with a final volume of 46.1 mL that was aged for 24 h at room temperature. ➢ Zirconia nanoparticles loaded sols (S1 and S2): first, GPTMS (9.52 g, 0.0403 mol) and AIP (2.4 g, 0.0118 mol) in a molar ratio of 3.4 were mixed in isopropanol 7.5 mL (0.0986 mol). The aqueous solution of cerium nitrate hexahydrate (0.67 g, 0.0015 mol in 6 g, 0.33 mol of water, 0.05 mol L^−1^) was added to the sol and stirred vigorously. Then, polyethylene glycol PEG 35000 (0.61 g) was diluted in distilled water to a final concentration of 20 g L^−1^ and added to the sol. The sol was aged for 24 h at room temperature, and then it was added to the zirconia suspensions containing 13.6 or 20.7 g in 25.9 mL of 1:25 water: isopropanol (v:v) to obtain S1 and S2 sols containing 30 wt% and 40 wt% of ZrO_2_ nanoparticles, respectively. ➢ Two architectured coatings were developed by dip-coating at a controlled withdrawal rate of 20 cm min^−1^. They were composed of a bi-layer coating that was developed using the sol and a zirconia-loaded mono-layer coating of S1 (30 wt%) and S2 (40 wt%) sols, respectively (named as C1 and C2 architectured coatings). The coating was dried for 1 h at 50 °C after each dip, followed by the final heating at 50 °C for 30 min and at 150 °C for 4 h.	❖ SEM morphologies showed that the thickness of the total architectured systems ranges between 5 μm and 10 μm for C1 and C2, respectively. Both coatings were composed of a nanocomposite outer layer of zirconia nanoparticles embedded in the alumino-silicated matrix with some aggregates of nanoparticles. ❖ The EIS results suggested that the impedance in the low-frequency range (0.01 Hz) for the coatings containing 40 wt% ZrO_2_ (C1) and 30 wt% ZrO_2_ (C2) was 10^6^ Ω cm^2^ and 10^7^ Ω cm^2^, respectively, which are significantly higher than that for the bare steel substrate (10^3^ Ω cm^2^). ❖ For the coated and then abraded samples, it was found that after 100 cycles of abrasion, impedance modulus values of the systems decreased from 10 Ω cm^2^ to 10^5^ Ω cm^2^. After 1000 cycles, the anticorrosion properties of the two coatings somewhat deteriorated due to the microstructural degradations.	[68]
✓ 3-glycidoxypropyltrimethoxysilane (GPTS)	➢ GPTS was hydrolyzed with deionized water (molar ratio of 3.5:0.7) using HCl as the catalyst. Zirconium-n-propoxide was complexed with methacrylic acid (MAA) under vigorous stirring. The zirconium-MAA complex was mixed with the hydrolyzed GPTS under stirring. Deionized water (0.07mol) was added, and the solution was stirred for 10 min to obtain the sol. The sol was diluted by 50% using 2-butoxyethanol and 1 wt% IRGACURE 184 (photoinitiator), followed by stirring for 15 min. The sol was then filtered through a 0.8 µm pore filter paper and used for coating. ➢ The mild steel samples were dipped in the solution using different withdrawal speeds: 0.5 mm/s, 1.0 mm/s and 2.0 mm/s. The coatings were UV-cured using a mercury lamp conveyorized UV curing unit, followed by thermal curing at 250 °C for 2 h in a vacuum drying oven (1 mbar).	❖ The coatings were deposited on mild steel, with and without plasma surface treatment, but both showed similar behavior with respect to their hydrophobicity, mechanical properties, such as pencil scratch hardness, and abrasion resistance. ❖ As suggested by both E_corr_ and i_corr_ data in PDP experiments, sol–gel coating considerably improved the corrosion resistance of mild steel. EIS results were consistent. However, the coating developed on plasma-treated mild steel showed superior performance than that on a substrate without plasma treatment.	[69]
✓ Tetraethylorthosilicate (TEOS)	➢ Silica sol: tetraethylorthosilicate (TEOS), ethanol and distilled water were mixed in a ratio of 4:5.5:90.5 (*v*/*v*). The solution was stirred for 10 min and stabilized over 4 days before use. ➢ Aluminum oxy-hydroxide sol: 12.5 wt% of aqueous NH_3_ was added dropwise to aluminum nitrate hexahydrate solution. The resulting precipitate was filtered and washed with distilled water and dispersed in distilled water to obtain a sol of 0.4 M concentration. ➢ Conversion coating: Mild steel (MS) coupons were dipped in silica sol and dried in ambient condition. Then, the silica-coated MS was dipped in aluminum oxy-hydroxide sol, dried in air, heated at 500 °C for 2 h and furnace cooled. After cooling, the coupons were cleaned in ethyl alcohol, to have the conversion coating, CcMS. ➢ Al_2_O_3_ sol and coatings: The Al_2_O_3_ sol was prepared by hydrolysis and polycondensation of aluminum isopropoxide (ACROS) and catalyzed by HNO_3_, as reported in the literature. The prepared sol was kept for 72 h with intermittent stirring to obtain clear sol before coating. The CcMS was dipped in alumina sol using a dip-coater at a constant withdrawal speed of 30 mm/min. The coated specimens were dried for 30 min in ambient atmosphere followed by heating at 250 °C for 15 min for removing the residual solvent. The dipping and drying process were repeated five times, followed by heating at 500 °C for 1 h.	❖ Surface of coatings heat-treated at 500 °C were fully covered with a dense vermicular structure. The surface appeared smooth, with featureless topography after sol–gel Al_2_O_3_ coating. ❖ The AFM suggested the silica sol coating (CcMS) was deposited as a layered structure comprising nanoparticles (>20 nm diameter), the layers being oriented in different directions. AFM of sol–gel coating suggested a uniform Al_2_O_3_ coating on CcMS; however, small dot-like features (i.e., agglomerates of 300–350 nm diameter) could be seen at higher magnifications). ❖ In PDP tests, i_corr_ of CcMS and CcMS/Al_2_O_3_ was found to be lower by 2 and 5 orders of magnitude, respectively, as compared to the bare mild steel (MS). The CcMS/Al_2_O_3_ coating shifted E_corr_ of MS, from −633 mV to +214 mV (i.e., comparable to E_corr_ of Al_2_O_3_). ❖ The defect-free and uniform sol–gel coating restricted the electrolyte ingress to the substrate, shifted E_corr_ towards the noble side and subsided the corrosion current density.	[72]
✓ Tetraethylorthosilicate (TEOS)	➢ TiO_2_–SiO_2_ sol was synthesized by dissolving 20 mL of titanium (IV) isopropoxide (TTIP) and 6 mL of TEOS in 130 mL of ethanol. Then, 6 mL of diethanolamine (DEA) was added. The mixture was stirred for 2 h and then aged at room temperature for 24 h. The resultant stable, transparent sol was used for single and multiple dip coatings on mild steel coupons. For multi-layer coatings, the film was allowed to dry at 120 °C for 30 min between two successive dippings. Finally, the films were fired at 410 °C for 30 min.	❖ The dry gel film thickness proportionally increased with the number of layers. Generally, the thickness of the fired film was presumed to be less compared to dry gel film. However, the fired film thickness for a single layer was somewhat higher. ❖ The fired two- or three-layer film followed the typical sol–gel coatings’ characteristics; however, fired films prevented fast growth of voluminous oxide.	[74]
✓ (3-glycidoxypropyl)-1,1,3,3-tetramethyldisiloxane (GTMD) ✓ Tetraethyl orthosilicate (TEOS)	➢ The silane precursors (GTMD and TEOS) were mixed for 30 min, followed by 5 mL of water addition that starts the hydrolytic conversion of the alkoxy groups into silanols. The mixture’s components were separated into two phases, though GTMD appeared to be sparingly miscible upon adding water, even with the alcohol diluent. To avoid such separation, a pre-stirred mixture of 5 mL water, 3 mL ethanol and 2 mL 0.05 N HNO_3_ was added to the silane mixture in drops. The amounts of silanes and solvents presented within this protocol were optimized for achieving homogeneous epoxy–silica hybrid (SG) coating suspension for coating on metals. The dilute acid mixture aided the simultaneous hydrolysis of the silane precursors and the ring-opening reaction of the epoxy functional group attached to the GTMD molecules. The unreacted methyl groups on GTMD provided some degree of hydrophobicity to the surface of the silica. The final SG hybrid coating suspension obtained after 2 weeks was relatively clear and stable for several days. ➢ Different concentrations of CeO_2_ pigment (0.25–2wt%) were added to the SG colloidal suspension (10 mL) under slow stirring. Usually, fillers are added in appropriate amounts to the coatings of this type for occupying free pore spaces/defects and for aiding the bridging of different layers within the coating structure, thereby reducing the chance of coating failure and delamination while enhancing surface adhesion. The precleaned and pre-treated metal substrates were dipped in modified and unmodified SG and immediately cured at 250 °C for 30 s and then at room temperature for one week.	❖ EIS measurements (Nyquist plots) revealed that the presence of CeO_2_ pigments in SG coating improved corrosion protection of mild steel due to the coating. The improvement is due to the formation of stable oxide/hydroxides of Ce within the coating network, as well as the pigment-enforced interconnecting oxide/hydroxide bridges CeO_2_ (1 wt%). Corrosion resistance of SG coating increased with CeO_2_ content of the coating, until 1 wt% CeO_2_ is reached. At greater CeO_2_ contents, the barrier properties start to drop (as also evidenced by the defects, detected by SEM), possibly because agglomeration of CeO_2_ starts to dominate. ❖ After the two-week exposure to 0.6 wt% NaCl plus 0.6 wt% (NH_4_)_2_SO_4_, SEM morphologies showed portions of the coatings with CeO_2_ (<1 wt%) to be still intact, whereas micro-cracks and exfoliation were observed in other coatings.	[77]
✓ Tetraethyl orthosilicate (TEOS) ✓ (3-aminopropyl) trimethoxysilane (APTES)	➢ Diluted silane precursors (0.1 M TEOS and 0.1 M APTES) were mixed with ethanol in the volume ratio of 3:1:2 (TEOS: APTES: ethanol). The mixture was doped with different concentrations of caffeine (i.e., 0, 25, 50, 75 and 100 ppm). The range of concentrations of caffeine was chosen based on its solubility and a predetermined screening test on inhibition in hybrid sol–gel matrices. Then, 1 mL of 0.1M HCl catalyst was added dropwise within the first hour of stirring to drive the simultaneous hydrolysis and condensation reactions. Finally, stirring of the mixture was continued at 30 ℃ for 24 h, which resulted in a transparent solution. Each hybrid sol–gel matrix was held still for one day before their application as coatings on mild steel.	❖ EIS results (Nyquist plots) showed an increase in corrosion resistance of mild steel in 3.5 wt% NaCl solution with the caffeine content (0–100 ppm) of the hybrid sol–gel coating. The charge transfer resistance (R_ct_) of the coating with 100 ppm caffeine was nearly 6.5 times higher than that of the bare mild steel. The improved corrosion resistance is attributed to the inhibitive effect of caffeine because of its ability of becoming adsorbed onto the steel surface and increasing the surface coverage of the coating. ❖ The trend of corrosion resistance determined by PDP and electrochemical noise measurement (ENM) was consistent with that in EIS results. ❖ The bare mild steel exposed to 3.5 wt% NaCl produced considerably lower-frequency current noise resistance (0.23 kΩ cm^2^) than mild steel coated with the hybrid sol–gel that contained 100 ppm caffeine (1.83 kΩ cm^2^).	[79]
✓ Tetraethyl orthosilicate (TEOS) ✓ (3-aminopropyl) triethoxysilane (APTES)	➢ For hybrid sol–gel preparation, 0.1M TEOS and 0.1M APTES were diluted with distilled water (with APTES/TEOS: water at 1:3). Ethanol (10 mL) was added with stirring. Then, 1 mL of 0.1M HCl was added and stirred for 24 h to produce a neat sol–gel matrix. Curcumin extract at concentrations of 25, 50, 75 and 100 ppm were added to 10 mL of ethanol to prepare a series of sol–gel matrices doped with the inhibitor. The formulation was stirred for 24 h, where 1 mL of 0.1 M HCl was added dropwise into the mixture. Mild steel coupons were dipped in the prepared sol–gel matrices for 24 h.	❖ PDP in 0.5M HCl showed considerably improved corrosion resistance of the mild steel due to the coating of silane doped with the ethanol extract of curcumin. The coating with highest curcumin content (100 ppm) performed the best, whereas the coating without the curcumin doping offered considerably inferior corrosion protection. EIS results were consistent with the findings of PDP.	[80]
✓ Tetraethyl orthosilicate (TEOS) ✓ 3-aminopropyltriethoxysilane (APTES)	➢ Precursors TEOS and APTES (diluted with water) and ethanol (99.5%) were used in a ratio of 3:1:2 (*v*/*v*). Simultaneously, different concentrations (0, 25, 50 and 75 ppm) of aqueous crude extract of span tea leaves were also added, and the pH was adjusted at 3 by adding 0.1 M HCl to catalyze the hydrolysis and condensation reactions. The formulation was continually stirred at ambient temperature for 24 h, followed by aging for 24 h that produced a clear transparent solution, with a solid content of 16 wt%. The sol–gel thus developed was applied as coatings on mild steel coupons by dipping them for 24 h, followed by curing for 24 h at room temperature.	❖ As evidenced by PDP results, the plain APTES-TEOS coating improved corrosion resistance (i_corr_, 3.1435 μA cm^−2^) of the uncoated mild steel (i_corr_, 6.2149 μA cm^−2^). However, a doping of the aqueous crude extract of span tea leaves achieved considerably improved corrosion resistance due to APTES-TEOS coating, with 75 ppm performing the best. ❖ The trend of corrosion resistance determined by PDP was consistent with that in electrochemical noise measurement (ENM) and EIS results. ❖ ENM showed the bare mild steel exposed to 3.5 wt% saline to produce considerably lower frequency current noise resistance (229.73 Ω cm^2^) than mild steel coated with the hybrid sol–gel coating that contained 75 ppm aqueous crude-extract doping (1318.55 Ω cm^2^). ❖ EIS data showed the R_ct_ value obtained from doping 75 ppm of aqueous crude extract into the sol–gel matrix to be six times higher than the bare mild steel substrate.	[81]
✓ Glycidoxypropyltrimethoxysilane (GPTMS) ✓ Tetraethyl orthosilicate (TEOS)	➢ The sol–gel recipe contained 7.5 mL GPTMS (0.1 M), 22.5 mL TEOS (0.1 M) and 10 mL of AR ethanol, to which 1 mL of 0.1 M HCl catalyst was added dropwise. Clitoria ternatea (CT) at concentrations of 25, 50, 75 and 100 ppm was added to the sol–gel doped with the inhibitor. Polished mild steel coupons were dipped into the sol–gel mixture for 24 h. The coating thickness was measured to be ~4 μm.	❖ As evidenced by PDP results in 0.5M HCl, the maximum corrosion inhibition was achieved in the case of sol–gel (GPTMS + TEOS) coating with the CT concentration of 75 ppm in the ethanol extract (EE) and water extract (W) (EE = 89.6% > WE = 85.6%). Neat sol–gel (GPTMS + TEOS) coating offered poor corrosion resistance, suggesting the effectiveness of CT doping. ❖ EIS results (Nyquist plots) corroborated PDP results.	[82]
✓ (3-glycidyloxypropyl) trimethoxysilane (GPTS)	➢ Synthesis and covalent functionalization of GO by GPTS: 1 g of graphite flakes was added to 120 mL concentrated H_2_SO_4_ with continuous stirring for 2 h. Then, 1 g NaNO_3_ was gradually added to the solution for 1 min, followed by adding 6 g KMnO_4_ to the solution over 30–45 min. The mixture was stirred for 72 h at ambient temperature. In an ice bath, the resultant solution was diluted with 300 mL of distilled water. After 30 min, H_2_O_2_ was added to the suspension, and the mixture was centrifuged and washed twice with 1M HCl and three times with distilled water, to obtain 200 mL water dispersion of graphene oxide (GO), i.e., 4.8 g/L. The aqueous GO suspension was subjected to a solvent exchange process to produce a fine GO dispersion in N, N dimethylformamide (DMF). The solvent exchange was carried out by adding DMF to the aqueous solution of GO, followed by sonication, centrifugation and removal of the supernatant. Surface functionalization of GO was carried out by mixing the GO (0.1 wt%) and GPTS (0.825 mL) under stirring and heating (100 °C for 3 h). The resulting black homogenous GPTS-functionalized GO (fGO) nanosheets were centrifuged and washed with absolute DMF. ➢ Fabrication of GO/PU and GO-GPTS/PU composites: The dispersions containing 100 mg GO and fGO nanosheets were individually mixed with 66 g acrylic resin for 15 min. As a curing agent, polyisocyanate hardener (Desmodur N75) was added to the GO/acrylic and fGO/acrylic dispersions. The ratio of acrylic resin to hardener was 2:1 *w/w*. ➢ Steel coupons were coated with the above materials by a film applicator. The coated samples were kept at room temperature for 24 h and then cured at 120 °C for 1 h.	❖ Salt spray tests: Corrosion protection due to the GO/PU and fGO/PU composites coating was assessed for up to 900 h. Blisters and corrosion spots were detected on the steel samples with GO/PU after 300 h and neat PU coatings after 600 h, i.e., the addition of neat GO to the PU matrix resulted in greater coating degradation/delamination than the neat PU coating. This was attributed to the role of GO nanosheets in increasing the coating hydrophilicity, and greater electrolytes diffuse through the coating due to the presence of many polar groups on the GO sheets, whereas, fGO/PU coating developed no corrosion spots and delamination for up to 600 h of exposure. Very occasional corrosion spots developed upon extending the exposure to 900 h. ❖ EIS tests: The significant decrease of |Z|_10 mHz_ for the neat PU with increasing the immersion time indicates the rapid deterioration in coating, allowing accelerated diffusion of corrosive electrolyte through the PU matrix and reaching the coating/metal interface to cause corrosion reaction.	[84]
✓ (3-aminopropyl) triethoxysilane (APTES) ✓ (3-glysidyloxypropyl) trimethoxysilane (GPTMS)	➢ Synthesis of silane-functionalized GO: 20 mg GO was dispersed in 40 mL DI water in a bath sonicator for 60 min. Then, 5 mL of 0.2 M toluene solution containing silane precursors (APTES and GPTMS) was added to the prepared GO solution. The formulated brownish solution was refluxed with continuous stirring at 70–80 °C for 24 h. The dark brown product of f-GO was washed with ethanol and DI water and dried at 50 °C. ➢ Preparation of nanocomposite coatings: f-GO powder was probe-sonicated for 1 h in an organic solvent. Then, it was added to the predetermined amount of polyamide hardener and sonicated for 5 min. The formed homogeneous dispersion of f-GO in polyamide hardener was stirred and heated at 70 °C to evaporate the solvent. The prepared mixture was added to the stoichiometric amount of epoxy resin (the stoichiometric mixing ratio for epoxy resin and hardener was 1:1 by weight), followed by mixing for 15 min. ➢ Subsequently, the prepared nanocomposites were placed in a vacuum oven for 1 h to degas and then applied by air spray on sandblasted mild steel samples. The coated samples were cured at room temperature and post-cured at 90 °C for 1 h.	❖ The open circuit potential (OCP) of coated steel shifted to more positive with the addition of nanofiller. The more noble value of OCP for nanocomposite coatings is due to the barrier effect of GO and f-GO nanofillers. The more positive OCP values for epoxy/f-GO compared to the other samples suggest higher corrosion protection ability. However, the OCP values gradually become more negative with increasing immersion time, which suggests the development of defects in the coating that allow diffusion of corrosive solution through the coating, causing corrosion of metal. ❖ EIS tests: Delamination appears in the case of neat epoxy coating in 7 days of immersion in 3.5 wt% NaCl solution, which caused a sudden and rapid decrease of |Z|_0.01 Hz_. The low corrosion resistance indicates deterioration of coating that caused high water absorption. The incorporation of nanofillers improved the corrosion resistance significantly, as suggested by the higher |Z|_0.01 Hz_ values that sustained during immersion in the corrosive for 28 days. The coating modified with f-GO showed higher |Z|_0.01 Hz_ than epoxy/GO coatings.	[85]
✓ (3-Aminopropyl) triethoxysilane (APTES)	➢ Synthesis of silane-functionalized GO: 20 mg GO was dispersed in 40 mL DI water in a bath sonicator for 60 min. Then, 5 mL of 0.2 M toluene solution containing silane precursor (APTES and GPTMS) was added to the prepared GO solution. The formulated brownish solution was refluxed with continuous stirring at 70–80 °C for 24 h. The dark brown product of f-GO was washed with ethanol and DI water and dried at 50 °C. ➢ GO and A-GO sheets were dispersed in acetone in a sonication bath for 1 h at room temperature. The prepared solution was mixed with polyamide hardener, employing sonication for 10 min to produce a homogeneous black suspension. The solution was heated at 50 °C while stirring to evaporate the solvent. The prepared mixture was mixed with a stoichiometric amount of epoxy resin (resin-to-hardener weight ratio of 1:1), using a high-shear mechanical mixer. Next, the prepared nanocomposites were placed in a vacuum oven for 1 h to degas and then applied by air spray on cleaned mild steel samples. The coated samples were cured at room temperature and post-cured at 90 °C for 1 h.	❖ EIS results revealed that steel with the epoxy/GO and epoxy/A-GO nanocomposite coatings possess significantly higher corrosion resistance than the steel with pure epoxy coating. The |Z|_0.01 Hz_ values for epoxy/A-GO coated samples are significantly higher than epoxy/GO coatings with the same loading, indicating excellent barrier properties of epoxy/A-GO. Incorporation of GO and A-GO nanosheets into the epoxy coating matrix significantly affects the corrosion protection. The corrosion resistance of coatings with respect to GO nanofiller addition improved in the sequence: pure epoxy < epoxy/0.05GO < epoxy/0.5GO < epoxy/0.3GO < epoxy/0.1GO. In terms of application of A-GO nanosheets in epoxy coatings, the corrosion resistance increases in the sequence of pure epoxy < epoxy/0.5A-GO < epoxy/0.05A-GO < epoxy/0.3A-GO < epoxy/0.1A-GO. For both epoxy/GO and epoxy/A-GO nanocomposite coatings, maximum corrosion protection is achieved at 0.1 wt% nanofiller addition.	[86]
✓ Isobutyl triethoxysilane (IBTS) ✓ Tetraethyl orthosilicate (TEOS)	➢ Isobutyl triethoxysilane and graphene oxide (IBTS/GO) and isobutyl triethoxysilane, tetraethyl orthosilicate and graphene oxide (IBTS/TEOS/GO) composite emulsions were prepared using the sol–gel method. The molar mass ratio of the two silanes was 1:1. Based on the most suitable solubility of GO, 0.3 wt% GO was chosen for this experiment. Ethanol and distilled water were used as the solvents, and pH was adjusted in the range of 4–6, using acetic acid, and vigorous stirring was employed for 5 h at 40 °C. When the silane was sufficiently hydrolyzed, the silane composite emulsions were carefully poured out. Carbon steel coupons were dipped into the GO-modified silane solution for 60 s and then removed slowly. The coated samples were cured at 80 °C for 2 h. The coating procedure was repeated three times to obtain enough coating thickness.	❖ EIS results (Nyquist plots) suggested corrosion resistance of IBTS/GO-coated mild steel to be improved by 50 times, to be more superior (|Z|, 100 kΩ cm^2^) than bare steel (|Z|, 2 kΩ cm^2^) in simulated concrete pore solution containing 3.5% NaCl, whereas IBTS/TEOS/GO coating provided only 5 times improvement (|Z|, 10 kΩ cm^2^). ❖ PDP results are consistent with EIS findings.	[87]
✓ Tetra Ethyl Ortho-Silane (TEOS) ✓ 3-Glycidaloxy Propyl Tri-methoxy Silane (GPTMS)	Preparation of graphene-based modified coatings: ➢ (rGO/CNT) coating: In 100 gm of epoxy resin, a mixture of 0.5 wt% of GPTMS and TEOS as silane agents by weight of the epoxy resin was mixed using a high shear homogenizer for 5 min to facilitate dispersion of fillers and surface modification. Then, rGO by 0.4 wt% and CNTs by 0.1 wt% of epoxy resin were incorporated into the resin–silane mixture and mixed using shear homogenizing at 20,000 rpm for 10 min. Adding rGO and CNTs to the prepared mixture improves barrier properties and strengthens the coating matrix. Then, the formed mixture is subjected to ultrasonication using a probe sonicator for 10 min in an ice bath to control the temperature of the matrix. Post-ultrasonication, the matrix solution was allowed to cool for 10 min. Next, the sample was subjected to constant stirring at 500 rpm using a mechanical stirrer, with the hardener (10:1) added gradually to the coating matrix for 5–10 min. The matrix was then coated on the plain steel bars with a paintbrush. The coated rebars were cured in the oven at 80 °C for 3 days before subjecting to accelerated corrosion or casting in concrete. ➢ (GO/CNT) coating: the procedure identical to that described above was followed by replacing rGO (0.4 wt% by epoxy resin) with GO. ➢ (FrGO) coating: Two solutions were prepared. In solution A, the functionalized rGO was prepared by mixing 46.92 gm of ethanol, 0.575 gm of distilled water and 2.5 gm of GPTMS using constant magnetic stirring for 10 min at room temperature. The rGO (2.5 g/l) was added to the solution, the pH of the mixture was adjusted to 4.5 using acetic acid, and the mixture was stirred using a magnetic stirrer for 2 h. Then, pH was increased to 11 by adding NaOH, and the solution was stirred for 1 h for the condensation reaction. Then, it was subjected to ultrasonication using a probe sonicator for 10 min followed by washing using a mixture of deionized water and ethanol (60:40 *w/w*). The washed functionalized rGO was extracted from the solution using vacuum filtration. Simultaneously, silane solution (solution B) was prepared by mixing 40 gm ethanol, 0.78 gm GPTMS, 1.72 gm TEOS and 7.5 gm distilled water using magnetic stirring. Functionalized rGO extracted from solution A was added into solution B, followed by continuous magnetic stirring for 1 h and ultrasonication for 10 min. Plain steel rebars were coated with the prepared solution using air spray having a pressure of 2 atm. After application of the first layer of solution, the rebar was dried in the oven at 120 °C for 10 min. This coating and drying process was repeated three times to ensure uniform distribution of rGO throughout the rebar section. After the third layer of coating application, samples were dried at 120 °C for 30 min. ➢ (FrGO + GO/CNT) coating: The base coat of functionalized rGO (called, FrGO) was used and applied to rebar samples, which were dried and further coated with formulation (GO/CNT) coating and cured.	Corrosion resistance: ❖ rGO/CNT coated rebars: In the accelerated corrosion tests, first, an increase in the corrosion current was observed after 36–40 days, which is attributed to the breakdown of the passive layer and corrosion initiation; this happened just in 6–7 days for the pure epoxy-coated bars. This showed a considerable improvement due to rGO/CNT incorporation into the epoxy coating. Moreover, further progress of corrosion was sluggish for rGO/CNT-coated steel. The improvement is due to a dense, impenetrable network formed in the epoxy, which blocks the entry of aggressive ions to the rebar. Since rGO is hydrophobic, it repels the saline solution and improves its corrosion barrier resistance compared to the PE coating. Even after 150 days of accelerated corrosion, no substantial increase in corrosion current was noticed. ❖ GO/CNT coated rebars: no increase or change in corrosion current was noticed even after 150 days of accelerated corrosion, indicating no corrosion initiation during the entire corrosion exposure period. ❖ FrGO coated rebars: Their behavior in the corrosive environment was similar to the control rebar. Within 2–3 h of corrosion exposure, the corrosion current reached 0.3A–0.34A, indicating the breakdown of the passive layer and initiation of corrosion. However, an essential difference between the two was that the FrGO-coated rebars did not corrode uniformly throughout the control rebar. Several pits were observed throughout the length. ❖ FrGO + GO/CNT coated rebars: With the inner layer of FrGO and GO/CNT as the top coating, corrosion spots appeared within seven days of exposure to the accelerated corrosion. The first increment in corrosion current was noticed after 22 days, indicating the breakdown of passive layer. The visual corrosion spots but not accompanied by any change in corrosion current are probably due to their being localized and small in size rather than deep pits.	[90]

## Data Availability

Data are contained within the article.
